# ﻿Taxonomic insights into the diversity of *Cloeon* Leach, 1815 (Ephemeroptera, Baetidae) in Thailand

**DOI:** 10.3897/zookeys.1266.176616

**Published:** 2026-01-05

**Authors:** Chayanon Noenrimnong, Chanaporn Suttinun, Nisarat Tungpairojwong, Boonsatien Boonsoong

**Affiliations:** 1 Animal Systematics and Ecology Speciality Research Unit (ASESRU), Department of Zoology, Faculty of Science, Kasetsart University, Bangkok 10900, Thailand Kasetsart University Bangkok Thailand; 2 Faculty of Veterinary Medicine, Chiang Mai University, Chiang Mai 50100, Thailand Chiang Mai University Chiang Mai Thailand; 3 Department of Biology, Faculty of Science, Khon Kaen University, Khon Kaen 40002, Thailand Khon Kaen University Khon Kaen Thailand; 4 Biodiversity Center Kasetsart University (BDCKU), Bangkok 10900, Thailand Biodiversity Center Kasetsart University (BDCKU) Bangkok Thailand

**Keywords:** Chorionic surface, DNA barcode, lentic habitats, mayfly

## Abstract

Four species of the genus *Cloeon* have previously been reported in Thailand: *C. bicolor* Kimmins, 1947, *C. bimaculatum* Eaton, 1885, *C. harveyi* (Kimmins, 1947) and *C. marginale* Hagen, 1858. However, since 1961, no systematic studies or investigations of this genus have been conducted in that country. This study reviews *Cloeon* Leach, 1815 in Thailand and investigates five species—*C. bengalense*, *C. bicolor*, *C. harveyi*, *C. rubellum*, and *C. viridulum*—based on morphological, molecular, and taxonomic analyses. Three species (*C. bengalense*, *C. rubellum*, and *C. viridulum*) are recorded for the first time in Thailand, and *C. rubellum* is described for the first time as a mature nymph and adult female. Additionally, the egg chorionic surface of all five species is described for the first time, and its use is demonstrated for species identification in Thailand. A key to the species of *Cloeon* in Thailand is provided based on the egg structure, mature nymph, and adult female and male.

## ﻿Introduction

The genus *Cloeon* was established by Leach (1815) and classified within the family Baetidae, one of the most diverse and widely distributed families of mayflies. The members of this genus are categorised as a swimmer group and are characterised by a relatively small body, elongated shape, antennae longer than the width of the head, slender legs, gills I–VI as doubled plates whereas gills VII are a single plate with three caudal filaments that assist in swimming (Leach 1815; Bouchard 2004). The nymphs of this genus feed on fine organic particles and are commonly found in lentic habitats, including ponds, lakes, pools, and temporary water bodies (Ying et al. 2021; Boonsoong 2022).

The genus *Cloeon* is widely distributed, occurring in North America, South America, Europe, Africa, Australia, the Pacific Islands and Asia, with approximately 74 species described worldwide (Sartori and Brittain 2015). It is found in Asian countries, such as India, Japan, Korea, Mongolia, China, and Thailand (Ying et al. 2021); however, the diversity of *Cloeon* species in Thailand remains unclear due to a lack of systematic studies (Boonsoong 2022). Previously, four species were reported in this country—*C. bicolor* Kimmins, 1947, *C. bimaculatum* Eaton, 1885, *C. harveyi* (Kimmins, 1947), and *C. marginale* Hagen, 1858 (Gillies 1949; Uéno 1961) — but no taxonomic investigations of this genus have been conducted in Thailand since 1961.

The limited data on species diversity and the taxonomy of this genus in Thailand pose challenges for further research in related fields. This paper aimed to study species diversity, review the taxonomy of the genus *Cloeon* in lentic habitats of Thailand, and associate the nymphal and imago stages based on morphological data. A further goal was to use molecular tools to expand research on this genus by sequencing the *COI* gene of known species to reconstruct the phylogenetic tree for this genus.

## ﻿Materials and methods

### ﻿Morphological observation

Specimens of the mayfly genus *Cloeon* were collected from lentic habitats, with a focus on urban and peri-urban areas. Imago specimens were collected directly in the field using light traps, while mature nymphs, identified by their dark wing pads, were collected using a D-frame net. Fully grown nymphs were reared in the laboratory using an earthen pot until they moulted into imago stages. All specimens were preserved in absolute ethyl alcohol. The specimens were examined and imaged using a Nikon SMZ800N stereomicroscope. Nymphal exuviae were mounted as permanent slides using Euparal and photographed with an Olympus BX51 compound microscope.

Eggs from *Cloeon* mayflies were processed using the critical point drying method with carbon dioxide (Quorum Technology Polaron Range CPD7501). The dried samples were mounted onto standard metal stubs, coated with gold (Polaron Range SC7620 Sputter Coater), and photographed using a scanning electron microscope (FEI Quanta 450). Measurements were taken using ImageJ software Java 1.8.0 (National Institutes of Health) (Schneider et al. 2012). Voucher specimens were deposited in the Aquatic Insects Collection of the Zoological Museum Kasetsart University (ZMKU) and Khon Kaen University (KKU).

### ﻿Molecular analysis

Each specimen was dissected, and total genomic DNA was extracted using the NucleoSpin® DNA purification kit (Macherey-Nagel, Germany), following the manufacturer’s instructions. A fragment of the mitochondrial cytochrome c oxidase subunit I (COI) gene was amplified using the primers LCO1490 and HCO2198 (Folmer et al. 1994). PCR conditions and protocols followed those described by Gattolliat et al. (2015). The PCR products were purified and sequenced by ATGC Co., Ltd (Thailand).

Sequence alignment, phylogenetic tree, and genetic distance processes were performed using MEGA 12 program (Kumar et al. 2024). The Tamura 3-Parameter Model and Gamma distribution with invariant sites (T92+G+I) were identified as the best-fit nucleotide substitution models and were executed using the maximum-likelihood method (ML) with 1000 bootstrap replicates. Nucleotide sequences obtained in this study are deposited in GenBank (Table 1). Other *Cloeon* sequences, which were obtained from GenBank, were *C. viridulum* (KR612257.1, KR612252.1, KR612249.1 from China), *C. dipterum* (MG784058.1 from USA, KY261135.1 from Germany, LC223583.1 from Japan), *C. navasi* (KR612258.1, KR612259.1 from China), *Cloeon* sp. (HM417037.1 from Thailand), and we used *Securiops primasia* (OQ573688.1 from Thailand) for the outgroup. The genetic distances between species were determined using p-distance.

**Table 1.** List of sequences of the genus *Cloeon* in Thailand.

Species	Locality	Code	Date	GenBank accession number
*C. bengalense*	Nakhon Ratchasima	NR01CV	29 Nov 2024	PX654150
	Kanchanaburi	KN01CV	22 Dec 2024	PX654151
	Bangkok	BM01CV	12 Jan 2025	PX654152
	Khon Kaen	KK01CV	01 Apr 2024	PX654153
	Khon Kaen	KK02CV	01 Apr 2024	PX654154
*C. bicolor*	Bangkok	BM01CB	26 Dec 2024	PX654155
	Nakhon Pathom	NP01CB	13 Jan 2025	PX654156
	Chachoengsao	CC01CB	05 Feb 2025	PX654157
	Khon Kaen	KK01CB	01 Apr 2024	PX654158
*C. harveyi*	Chiang Mai	CM01CH	07 Jan 2025	PX654159
	Bangkok	BM01CH	29 Nov 2024	PX654139
	Chachoengsao	CC01CH	29 Dec 2024	PX654160
	Khon Kaen	KK01CH	01 Apr 2024	PX654161
	Khon Kaen	KK02CH	01 Apr 2024	PX654162
	Khon Kaen	KK03CH	01 Apr 2024	PX654140
*C. rubellum*	Chachoengsao	CC01CR	29 Dec 2024	PX654163
	Bangkok	BM01CR	02 Feb 2025	PX654164
	Bangkok	BM02CR	04 Jan 2025	PX654165
	Khon Kaen	KK01CR	01 Apr 2024	PX654166
	Khon Kaen	KK02CR	01 Apr 2024	PX654167
	Khon Kaen	KK03CR	01 Apr 2024	PX654168
	Khon Kaen	KK04CR	01 Apr 2024	PX654169
	Khon Kaen	KK05CR	01 Apr 2024	PX654170
	Khon Kaen	KK06CR	01 Apr 2024	PX654171
	Khon Kaen	KK07CR	01 Apr 2024	PX650272
*C. viridulum*	Kanchanaburi	KN01CD	05 July 2025	PX650275

Species delimitation was performed using the distance-based Assemble Species by Automatic Partitioning (ASAP) application (Puillandre et al. 2021), applying the Kimura 2-parameter (K2P) model (Kimura 1980) under default parameter settings. We followed all guidelines of the Animal Ethics Committee of Kasetsart University (approval no. ACKU68-SCI-017) for collecting the mayfly specimens.

## ﻿Taxonomic accounts

**Family Baetidae**

**Genus *Cloeon* Leach, 1815**

### ﻿ Cloeon bengalense

Taxon classificationAnimaliaEphemeropteraBaetidae

Kimmins, 1947

C6283AC3-18BA-500D-A292-34AB9C79A09D

Figs 1A–C, 6A, B, 8A, B, 10A, B, 13A, B, 15A, B, 18A–D, 19A–F, 20A–F, 27C, 28E, 29D

Cloeon bengalense Kimmins: 95, figs 3, 7, 11 (original description, male and female).
Cloeon bengalense : Kimmins 1971: 310 (list). Hubbard and Peters 1978: 8 (list). Hubbard and Srivastava 1984: 3 (list). Marcus 2019: 85, figs 1–4 (record).

#### Material examined.

• 4 nymphs, 5 female subimagos, 3 female imagos, **Bangkok Prov.**, Luang Suwannawajokkasikij 100^th^ Year Park, 13°50'54.5"N, 100°34'15.1"E, 1.VIII.2024, C. Noenrimnong leg. (ZMKU); • 4 nymphs, 1 male subimago, **Bangkok Prov.**, Varunawan Park, 13°50'51.1"N, 100°33'45.9"E, 25.X.2024, B. Boonsoong & C. Noenrimnong leg. (ZMKU); • 8 nymphs, 1 female subimago, 2 male imagos, 3 female imagos, **Bangkok Prov.**, Information Building 50 Years, 13°51'11.6"N, 100°34'11.9"E, 12.I.2025, C. Noenrimnong leg. (ZMKU); • 7 male subimagos, 1 female subimago, **Lop Buri Prov.**, Huai Som, 14°51'52.3"N, 100°51'28.1"E, 19.I.2025, A. Wongyam leg. (ZMKU); • 7 nymphs, 1 female imago, **Chachoengsao Prov.**, Sao Cha-Ngok, 13°41'43.1"N, 101°09'13.5"E, 8.XI.2024, B. Boonsoong leg. (ZMKU); • 3 nymphs, **Prachin Buri Prov.**, Kabin Buri, 13°59'04.3"N, 101°45'11.4"E, 15.V.2025, B. Boonsoong leg. (ZMKU); • 4 nymphs, 1 male subimago, 2 male imagos, 1 female imago, **Nakhon Ratchasima Prov.**, Chateau de Khaoyai, 14°30'48.6"N, 101°27'06.0"E, 29.XI.2024, B. Boonsoong leg. (ZMKU); • 2 nymphs, **Maha Sarakham Prov.**, Nong Bua Kaeo, 15°31'56.4"N, 103°18'26.0"E, 16.V.2025, B. Boonsoong leg. (ZMKU); • 3 nymphs, **Buri Ram Prov.**, Mafueang Phutthaisong, 15°29'54.0"N, 103°02'58.2"E, 16.V.2025, B. Boonsoong leg. (ZMKU); • 14 nymphs, **Khon Kaen Prov.**, Sithan Lake KKU, 16°26'41.1"N, 102°48'55.0"E, 1.IV.2024, N. Tungpairojwong leg. (KKU); • 22 nymphs, **Khon Kaen Prov.**, Fisheries Pond KKU, 16°27'31.5"N, 102°48'38.3"E, 1.IV.2024, N. Tungpairojwong leg. (KKU); • 3 nymphs, 1 male subimago, 1 female imago, **Khon Kaen Prov.**, Khot swamp, 16°26'06.1"N, 102°48'09.0"E, 17.V.2025, B. Boonsoong leg. (ZMKU); • 5 nymphs, 1 female imago, **Mukdahan Prov.**, Nut Pob Rim Kong Mukdahan, 16°31'53.7"N, 104°44'05.0"E, 18.V.2025, B. Boonsoong leg. (ZMKU); • 1 female imago, **Chiang Mai Prov.**, Faculty of Veterinary Medicine CMU, 18°45'32.2"N, 98°56'24.8"E, 21.X.2025, C. Suttinun leg. (ZMKU); • 1 female imago, **Kanchanaburi Prov.**, Kwai Noi River, 14°12'07.7"N, 99°03'36.4"E, 22.XII.2024, B. Boonsoong & C. Noenrimnong leg. (ZMKU); • 6 nymphs, 1 male subimago, 1 female imago, **Kanchanaburi Prov.**, The River Kwai Bridge Resort, 14°02'26.7"N, 99°30'26.6"E, 05.VII.2025, B. Boonsoong leg. (ZMKU); • 3 male imagos, **Ratchaburi Prov.**, Bua Resort Suanphueng, 13°31'30.0"N, 99°14'40.3"E, 15.III.2025, C. Noenrimnong leg. (ZMKU).

#### Descriptions

**(in alcohol**)**. Female imago** (Fig. 6A). See Kimmins (1947) for original description. The female imagos of this species can be distinguished by the pale green to yellowish-green head, which bears a pale green midline flanked by two dark brown longitudinal stripes covering most of the dorsal surface (Fig. 18A). The thorax is similarly coloured, with a pale green to yellowish-green ground colour and a pale midline flanked by two continuous dark brown longitudinal stripes extending from the head to the abdomen (Fig. 18A). The abdominal terga are green to yellowish-green, with dark brown to dark reddish-brown pigmentation. Terga II–IX have a pale midline flanked by a pair of pale spots or drop-shaped stripes; the lateral margins of anterior terga exhibit dark reddish-brown spots or short stripes, with those on segments III and VI extending almost to the lateral margins (Fig. 13A). The abdominal sterna exhibit dark reddish-brown spots along the lateral margins of segments II–VIII (Fig. 15A). The wings are hyaline, with dark brown venation in the costal and subcostal areas (Fig. 10A).

**Male imago** (Fig. 6B). See Kimmins (1947) for original description.

The male imagos are recognised by their orange-yellow compound eyes with olive-green lower portions (Fig. 8B). The abdominal terga are translucent white, with segments III and VI bearing dark reddish-purple stripes extending nearly to the lateral margins; smaller paired stripes occur on segments II and V. Segments VIII–X are variably coloured from orange to dark brown (Fig. 13B). The abdominal sterna of segments VIII and IX are white, while sternum X ranges from orange to dark brown (Figs 15B, 18B). The wings are hyaline (Fig. 10B).

**Mature nymph (in alcohol)**: Body length 5.66–5.67 mm, terminal filaments 3.44–3.49 mm.

**Figure 1.**
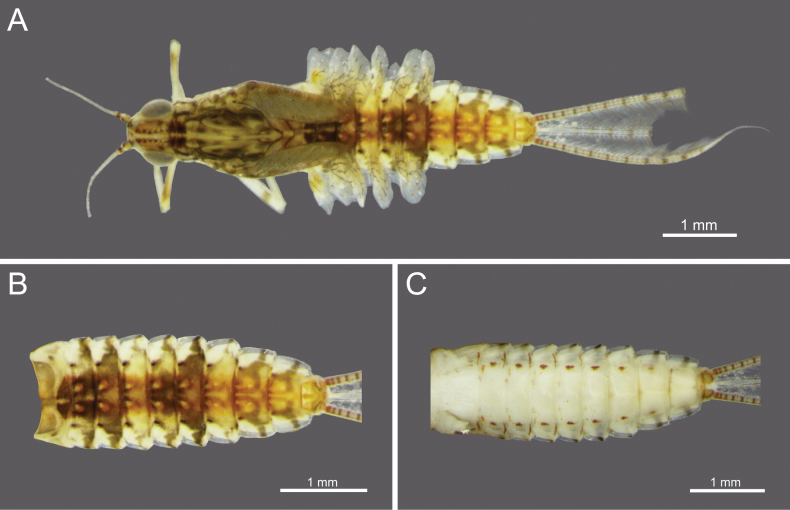
*Cloeon bengalense*, mature nymph morphology: **A.** Female mature nymph (dorsal view); **B.** Terga I–X; **C.** Sterna I–X.

***Head***: Two rows of irregular brown spots (Fig. 1A). Antennae mostly brown, with darker colouration at the base (Fig. 19A).

***Mouthparts***: i). Mandibles: Molar area contains densely distributed molar teeth. Lateral margins have sparsely distributed hair-like setae. Large prostheca, inner incisors, and outer incisors (Fig. 19B, C). ii). Labrum: Dorsal surface and half margins covered with hair-like setae. More than half of the surface contains sparsely distributed hair-like setae, with tufts of setae on either side of the anterior notch (Fig. 19D). iii). Maxillary Palp: Segments I and II with sparsely distributed hair-like setae on the surface, while segment III with more widely distributed hair-like setae (Fig. 19E). iv). Labial Palpi: Segment I longer than segments II and III. Segments I and II with sparsely distributed hair-like setae, while segment III with hair-like setae densely distributed around the terminal margin and longer and denser than those on other segments. Glossae and para-glossae with longer setae on the margins (Fig. 19F).

***Thorax***: Irregular brown markings (Fig. 1A).

***Legs***: Femora of all legs pale but with brown stripes near the apex and base, short setae on the surface and both margins. Tibiae and tarsi with brown bands near their bases, with short, sparsely distributed setae on the surface and dense, medium-sized setae along the inner margins (Fig. 20A–C). Base of the claws (foreleg) expanded, with the basal half containing two rows of spines with gradation size (Fig. 20D).

***Abdominal terga***: Green to yellowish-green, uniform dark brown to dark reddish-brown. Segments II to IX, anterior part with a pale midline flanked by a pair of pale spots or drop-shaped stripes. Segments II and V may appear darker, while segments IV and VII may appear slightly paler but still retain the patterns. More than half of the lateral margin of segment VIII has 6–8 spines; whole of the lateral margin of segment IX has 6–11 spines (Figs 1A, B, 20E).

***Abdominal sterna***: Green to yellowish-green, segments VII to IX with red to pale red spots near the middle posterior part, while segment X appears darker (Fig. 1C).

***Caudal Filaments***: Each segment has a ring of spines at joints. Cerci with long setae along the inner margins and large, long spines on the outer margins. Terminal filament shorter than cerci, and long setae along both margins (Figs 1A, 20F).

**Egg.** Width 43.37 μm; height 68.12 μm; filament thickness ranging from 0.154 to 0.173 μm. Oval-shaped; surface uniformly covered with dense, long filamentous structures (Figs 18C, D).

#### Distribution.

Northern Thailand (Chiang Mai province); northeastern Thailand (Buri Ram, Khon Kaen, Maha Sarakham, Mukdahan, and Nakhon Ratchasima provinces); central Thailand (Bangkok and Lop Buri provinces); eastern Thailand (Chachoengsao and Prachin Buri provinces); western Thailand (Kanchanaburi and Ratchaburi provinces).

#### Remarks.

The adults of *Cloeon bengalense* were described by Kimmins (1947) based on materials from West Bengal (India). This species has also been recorded in Singapore from the adult stage. Ours is the first record of this species in Thailand. In this study, we provide the first description of the egg structure and nymphal stage of *C. bengalense*. Additionally, we describe the smaller paired stripes that occur on segment V of the male imago, thereby supplementing the original description by Kimmins (1947).

### ﻿ Cloeon bicolor

Taxon classificationAnimaliaEphemeropteraBaetidae

Kimmins, 1947

1E3B68F4-D2F5-5FA7-A80D-E4506B763308

Figs 2A–C, 7C, 8C, D, 10C, D, 13C, D, 15C, D, 21A–D, 27D, 28C, 29C

Cloeon bicolor Kimmins, 1947: 97, figs 4, 8, 12 (original description, male and female).
Cloeon bicolor Kimmins: Gillies 1949: 173. Kimmins 1971: 311 (list). Hubbard and Peters 1978: 8. Hubbard and Srivastava 1984: 3. Selvakumar et al. 2016: 653 (data on DNA). Kubendran et al. 2017: 615. Ying et al. 2021: 2, figs 1A, B, 2, 3A, 3E, 3I, 3M, 4, 5A, B, 6A, B, 7A–C, 8A, B (nymph first description). Kluge 2022: 161, 166. Kubendran et al. 2022: 398, figs 1–17 (record).

#### Material examined.

• 1 nymph, 1 female imago, **Bangkok Prov.**, Luang Suwannawajokkasikij 100^th^ Year Park, 13°50'54.5"N, 100°34'15.1"E, 1.VIII.2024, C. Noenrimnong leg. (ZMKU); • 1 female subimago, 2 male imagos, **Bangkok Prov.**, Charn at the Avenue, 13°53'40.8"N, 100°33'27.8"E, 26.XI.2024, S. Kwanboon leg. (ZMKU); • 1 male subimago, 1 female subimago, **Lop Buri Prov.**, Huai Som, 14°51'52.3"N, 100°51'28.1"E, 19.I.2025, A. Wongyam leg. (ZMKU); • 2 female imagos, **Chanthaburi Prov.**, Laemsing, 12°29'05.0"N, 102°04'10.2"E, 29.XII.2024, C. Noenrimnong leg. (ZMKU); • 1 male imago, **Nakhon Ratchasima Prov.**, Non Sung, 15°14'05.5"N, 102°22'40.6"E, 19.V.2025, B. Boonsoong leg. (ZMKU); • 1 nymph, **Nakhon Ratchasima Prov.**, Motorway M6, 14°53'09.8"N, 101°37'17.5"E, 19.V.2025, B. Boonsoong leg. (ZMKU); • 1 female imago, **Buri Ram Prov.**, Mafueang Phutthaisong, 15°29'54.0"N, 103°02'58.2"E, 16.V.2025, B. Boonsoong leg. (ZMKU); • 2 nymphs, **Khon Kaen Prov.**, Sithan Lake KKU, 16°26'41.1"N, 102°48'55.0"E, 1.IV.2024, N. Tungpairojwong leg. (KKU); • 7 nymphs, **Khon Kaen Prov.**, Fisheries Pond KKU, 16°27'31.5"N, 102°48'38.3"E, 1.IV.2024, N. Tungpairojwong leg. (KKU); • 4 male imagos, **Roi Et Prov.**, Na Pho Subdistrict, 15°57'48.8"N, 103°34'08.2"E, 10.VII.2025, W. Singsanan leg. (ZMKU); • 4 nymphs, 1 female subimago, **Ubon Ratchathani Prov.**, Ubon Ratchathani University, 15°07'06.1"N, 104°54'09.4"E, 22.VIII.2025, B. Boonsoong leg. (ZMKU); • 1 female imago, **Bangkok Prov.**, Chalermprakiat 55^th^ Year Park, 13°51'17.0"N, 100°34'27.7"E, 1.VIII.2024, C. Noenrimnong leg. (ZMKU); • 1 male imago, **Nakhon Pathom Prov.**, Chawalun Resort, 13°56'31.7"N, 100°03'12.7"E, 13.I.2025, S. Kwanboon leg. (ZMKU); • 3 female imagos, **Chachoengsao Prov.**, Sao Cha-Ngok, 13°41'43.1"N, 101°09'13.5"E, 8.XI.2024, B. Boonsoong leg. (ZMKU).

#### Description

**(in alcohol). Female imago.** See Kimmins (1947) for original description.

The female imagos are characterised by a yellow head, with a pale-yellow midline flanked by two dark brown longitudinal stripes across the central region (Fig. 21A). The thorax is yellow, with a yellow midline bordered by two narrow, long, pale brown longitudinal stripes extending from the head (Fig. 21A). The abdominal terga are yellow, with uniformly brown to dark reddish-brown pigmentation. Terga II–IX have a pale midline flanked by a pair of pale spots or drop-shaped markings, while the lateral margins of each anterior segment bear dark reddish-brown spots or stripes (Fig. 13C). The abdominal sterna display dark reddish-brown stripes along the lateral margins of segments II–VIII (Fig. 15C). The wings are hyaline, with a pale-yellow stripe in the costal area and a dark brown stripe in the subcostal area (Fig. 10C).

**Male imago** (Fig. 7C). See Kimmins (1947) for original description. The male imagos of this species can be identified by their hyaline wings (Fig. 10D), which lack strong pigmentation except for a pale-yellow stripe in the costal area and a dark brown stripe in the subcostal area. The abdominal terga are translucent white, with segments II–VII showing similar patterns to those of the female but narrower, featuring faint reddish-brown spots along the lateral margins. Terga VIII–X are orange to dark orange in colouration (Fig. 13D). The abdominal sterna of segments VIII–X are white and unpigmented (Figs 13D, 15D, 21B). The compound eyes are orange to orange-yellow, with olive-green lower portions (Fig. 8D).

**Figure 2.**
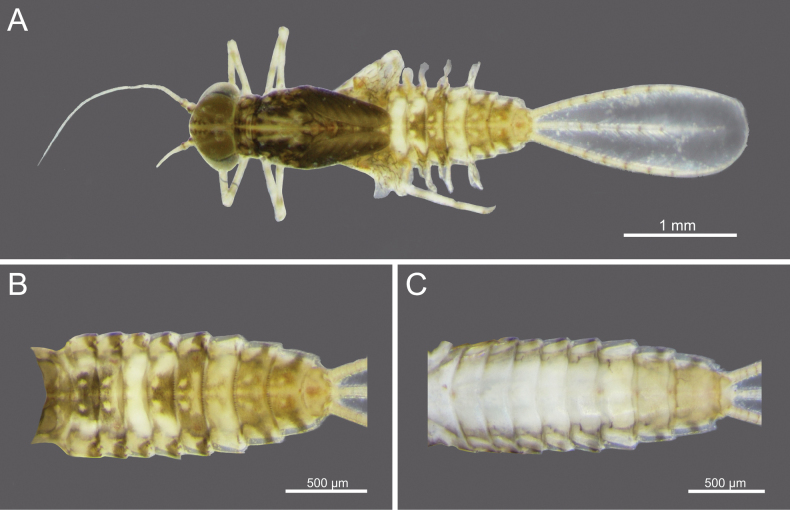
*Cloeon bicolor*, mature nymph morphology: **A.** Male mature nymph (dorsal view); **B.** Terga I–X; **C.** Sterna I–X.

**Nymph.** See Ying et al. (2021) for first description. The mature nymphs possess a head with two rows of irregular brown spots (Fig. 2A). The abdominal terga are dark green to brown, with uniformly dark brown to reddish-brown colouration. Terga II–IX bear a pale midline flanked by a pair of pale spots or drop-shaped marks. Segments II and V may appear darker, with interruptions in the midline, while segments IV and VII are comparatively paler (Fig. 2A, B). The abdominal sternum X lacks dark pigmentation (Fig. 2C).

**Egg.** Width 71.61 μm; height 95.98 μm; granules ranging from 0.086–0.118 μm. Oval to nearly round; surface covered with granules of similar size, distributed unevenly. Some areas of surface appear smoother, with more widely spaced granules (Fig. 21C, D).

#### Distribution.

Northeastern Thailand (Buri Ram, Khon Kaen, Nakhon Ratchasima, Roi Et, and Ubon Ratchathani provinces); central Thailand (Bangkok, Lop Buri, and Nakhon Pathom provinces); eastern Thailand (Chachoengsao and Chanthaburi provinces).

#### Remarks.

The adults of *Cloeon bicolor* were described by Kimmins (1947) based on materials from Bengal (India). This species was also recorded from China and the nymph was described by Ying et al. (2021). In this study, we provide the first description of the egg structure of *C. bicolor*.

### ﻿ Cloeon harveyi

Taxon classificationAnimaliaEphemeropteraBaetidae

(Kimmins, 1947)

6B344D07-3B78-5582-8FAF-82397AF0A599

Figs 3A–C, 6C, D, 9A, B, 11A, B, 14A, B, 16A, B, 22A–D, 27A, 28B, 29A

Procloeon harveyi Kimmins, 1947: 94, figs 2, 6, 10 (original description, male, female).
Procloeon harveyi Gillies 1949: 176 (record). Kimmins 1971: 314 (list). Hubbard and Peters 1978: 11. Hubbard and Srivastava 1984: 3. Kandibane et al. 2007: 743. Mukherjee et al. 2012: 55, figs 1–9 (nymph first description).
Cloeon harveyi (Kimmins, 1947): Müller-Liebenau and Hubbard 1986: 538. Hubbard 1986: 248.

#### Material examined.

• 99 nymphs, 2 male subimagos, 52 male imagos, 21 female imagos, **Bangkok Prov.**, Luang Suwannawajokkasikij 100^th^ Year Park, 13°50'54.5"N, 100°34'15.1"E, 1.VIII.2024, C. Noenrimnong leg. (ZMKU); • 14 nymphs, 6 male imagos, 4 female imagos, **Bangkok Prov.**, Chalermprakiat 55^th^ Year Park, 13°51'17.0"N, 100°34'27.7"E, 1.VIII.2024, C. Noenrimnong leg. (ZMKU); • 20 nymphs, 1 female subimago, 11 male imagos, 6 female imagos, **Bangkok Prov.**, Varunawan Park, 13°50'51.1"N, 100°33'45.9"E, 25.X.2024, B. Boonsoong & C. Noenrimnong leg. (ZMKU); • 21 nymphs, 9 male imagos, 7 female imagos, **Bangkok Prov.**, Information Building 50 Years, 13°51'11.6"N, 100°34'11.9"E, 12.I.2025, C. Noenrimnong leg. (ZMKU); • 13 nymphs, 16 male subimagos, 16 female subimagos, 4 male imagos, 1 female imago, **Bangkok Prov.**, Emarica Academy, 13°50'39.8"N, 100°33'54.4"E, 2.II.2025, S. Kwanboon & C. Noenrimnong leg. (ZMKU); • 1 male subimago, **Lop Buri Prov.**, Huai Som, 14°51'52.3"N, 100°51'28.1"E, 19.I.2025, A. Wongyam leg. (ZMKU); • 25 nymphs, 5 male subimagos, 2 female subimagos, 28 male imagos, 6 female imagos, **Chachoengsao Prov.**, Sao Cha-Ngok, 13°41'43.1"N, 101°09'13.5"E, 8.XI.2024, B. Boonsoong leg. (ZMKU); • 2 nymphs, 2 male imagos, 6 female imagos, **Nakhon Nayok Prov.**, Wang Takrai, 14°20'09.3"N, 101°18'22.1"E, 8.III.2025, B. Boonsoong leg. (ZMKU); • 4 nymphs, **Khon Kaen Prov.**, Sithan Lake KKU, 16°26'41.1"N, 102°48'55.0"E, 1.IV.2024, N. Tungpairojwong leg. (KKU); • 17 nymphs, **Khon Kaen Prov.**, Fisheries Pond KKU, 16°27'31.5"N, 102°48'38.3"E, 1.IV.2024, N. Tungpairojwong leg. (KKU); • 1 nymph, **Ubon Ratchathani Prov.**, Ubon Ratchathani University, 15°07'06.1"N, 104°54'09.4"E, 22.VIII.2025, B. Boonsoong leg. (ZMKU); • 2 nymphs, 4 male imagos, 4 female imagos, **Chiang Mai Prov.**, Mae Jam, 18°29'47.8"N, 98°21'38.3"E, 15.XII.2024, B. Boonsoong leg. (ZMKU); • 1 male imago, 7 female imagos, **Chiang Mai Prov.**, San Phak Wan, 18°42'54.3"N, 98°56'59.1"E, 7.I.2025, C. Suttinun leg. (ZMKU); • 13 nymphs, 9 male imagos, 4 female imagos, **Ratchaburi Prov.**, Bua Resort Suanphueng, 13°31'30.0"N, 99°14'40.3"E, 15.III.2025, C. Noenrimnong leg. (ZMKU); • 4 nymphs, 1 female imago, **Phetchaburi Prov.**, Centara Life Cha-Am Beach Resort, 12°44'03.3"N, 99°57'52.3"E, 3.VII.2025, B. Boonsoong leg. (ZMKU); • 5 nymphs, **Trang Prov.**, Tham Khao Chang Hai, 7°35'08.5"N, 99°39'54.2"E, 11.IV.2025, P. Numuan leg. (ZMKU).

#### Description

**(in alcohol). Male imago** (Fig. 6D). See Kimmins (1947) for original description.

The male imagos of this species can be identified by their transparent wings (Fig. 11B), with distinct dark brown to black pigmentation in the pterostigma area and pale brown markings along the costal and subcostal regions. The abdominal terga are translucent white, with prominent reddish-brown markings on segments II, III and VI; segment VII has paler patterns, and segments VIII–IX are reddish brown (Fig. 14B). The abdominal sterna of segments VIII–IX and the posterior portion of segment VII are white, marked with reddish-brown striped patterns (Figs 16B, 22B). The compound eyes are yellowish-brown with olive-green lower portions (Fig. 9B).

**Female imago** (Fig. 6C). See Kimmins (1947) for original description. The female imagos can be distinguished by a cream-white head, bordered apically with a rust-coloured margin (Fig. 22A). The thorax is cream-white, bearing a pair of midline dark brown stripes narrowing anteriorly and flanked by white lines (Fig. 22A). The abdominal terga are cream-white, with prominent reddish-brown markings on segments II, III, and VI, and posterior parts of terga I–IX bearing a pair of reddish-brown stripes (Fig. 15A). The abdominal sterna exhibit two reddish-brown longitudinal stripes along the abdomen that fuse apically (Fig. 16A). The wings are hyaline, with clearly pigmented dark brown to black markings in the pterostigma area, and pale brown markings in the costal and subcostal regions (Fig. 11A).

**Figure 3.**
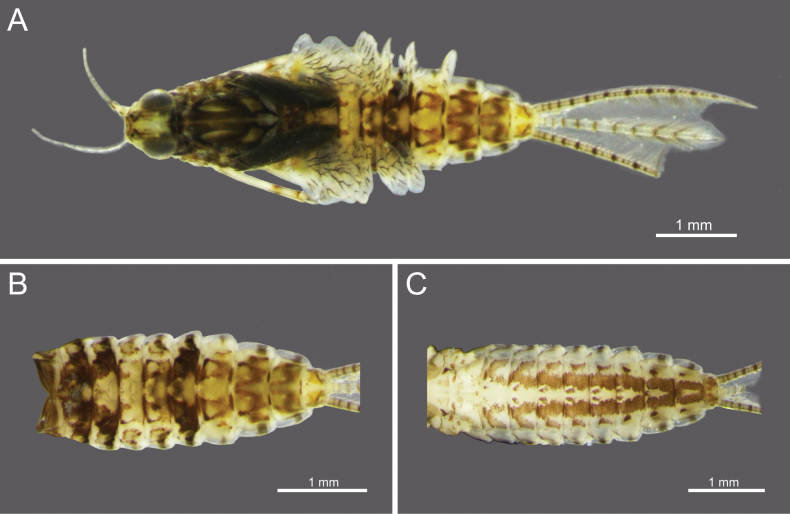
*Cloeon harveyi*, mature nymph morphology: **A.** Female mature nymph (dorsal view); **B.** Terga I–X; **C.** Sterna I–X.

**Nymph.** See Mukherjee et al. (2012) for first description and Ying et al. (2021). The mature nymphs resemble the imago in colouration. The head shows a similar pattern (Fig. 3A), and abdominal terga bear reddish-brown markings on segments II, III, and VI, consistent with the adult stage (Figs 3A, B). The abdominal sterna display two reddish-brown longitudinal stripes that merge on segments VIII–IX (Fig. 3C).

**Egg.** Width 59.82 μm; height 96.78 μm; granules ranging from 0.085–0.101 μm. Oval-shaped; surface covered with granules of consistent size and distribution, high density (Fig. 22C, D).

#### Distribution.

Northern Thailand (Chiang Mai province); northeastern Thailand (Khon Kaen and Ubon Ratchathani provinces); central Thailand (Bangkok, Lop Buri, and Nakhon Nayok, provinces); eastern Thailand (Chachoengsao province); western (Ratchaburi and Phetchaburi provinces); southern Thailand (Trang province).

#### Remarks.

The adults of *Cloeon harveyi* were described by Kimmins (1947) based on materials from Bengal (India). This species is also recorded from China (south, Hong Kong), Malaysia, and Thailand. The nymph was described by Mukherjee et al. (2012) and Ying et al. (2021). In this study, we provide the first description of the egg structure of *C. harveyi*.

### ﻿ Cloeon rubellum

Taxon classificationAnimaliaEphemeropteraBaetidae

Navás, 1923

38C6997C-932D-558B-B0DA-CB682344E783

Figs 4A–C, 7A, B, 9C, D, 11C, D, 14C, D, 16C, D, 23A–D, 24A–F, 25A–F, 27B, 28A, 29E

Cloeon rubellum Navás, 1923: 3 (original description, male).
Cloeon rubellum : Lestage 1929: 99 (key to species). Hubbard and Pescador 1978: 92. Alba-Tercedor and Peters 1985: 221.

#### Material examined.

• 11 nymphs, 1 female imago, **Bangkok Prov.**, Luang Suwannawajokkasikij 100^th^ Year Park, 13°50'54.5"N, 100°34'15.1"E, 1.VIII.2024, C. Noenrimnong leg. (ZMKU); • 10 nymphs, 1 male subimago, 2 female subimagos, 7 male imagos, 2 female imagos, **Bangkok Prov.**, Chalermprakiat 55^th^ Year Park, 13°51'17.0"N, 100°34'27.7"E, 1.VIII.2024, C. Noenrimnong leg. (ZMKU); • 2 nymphs, **Bangkok Prov.**, Varunawan Park, 13°50'51.1"N, 100°33'45.9"E, 25.X.2024, B. Boonsoong & C. Noenrimnong leg. (ZMKU); • 4 male subimagos, 12 female subimagos, **Bangkok Prov.**, Charn at the Avenue, 13°53'40.8"N, 100°33'27.8"E, 26.XI.2024, S. Kwanboon leg. (ZMKU); • 3 nymphs, **Bangkok Prov.**, Information Building 50 Years, 13°51'11.6"N, 100°34'11.9"E, 12.I.2025, C. Noenrimnong leg. (ZMKU); • 3 nymphs, 4 male subimagos, 2 female subimagos, 1 male imago, 2 female imagos, **Bangkok Prov.**, Emarica Academy, 13°50'39.8"N, 100°33'54.4"E, 2.II.2025, S. Kwanboon & C. Noenrimnong leg. (ZMKU); • 2 female subimagos, **Lop Buri Prov.**, Huai Som, 14°51'52.3"N, 100°51'28.1"E, 19.I.2025, A. Wongyam leg. (ZMKU); • 2 nymphs, 18 male imagos, 10 female imagos, **Chachoengsao Prov.**, Sao Cha-Ngok, 13°41'43.1"N, 101°09'13.5"E, 8.XI.2024, B. Boonsoong leg. (ZMKU); • 2 nymphs, 1 male imago, **Chanthaburi Prov.**, Laemsing, 12°29'05.0"N, 102°04'10.2"E, 29.XII.2024, C. Noenrimnong leg. (ZMKU); • 2 male imagos, **Nakhon Ratchasima Prov.**, Non Sung, 15°14'05.5"N, 102°22'40.6"E, 19.V.2025, B. Boonsoong leg. (ZMKU); • 1 nymph, 1 male imago, **Buri Ram Prov.**, Mafueang Phutthaisong, 15°29'54.0"N, 103°02'58.2"E, 16.V.2025, B. Boonsoong leg. (ZMKU); •3 nymphs, **Khon Kaen Prov.**, Sithan Lake KKU, 16°26'41.1"N, 102°48'55.0"E, 1.IV.2024, N. Tungpairojwong leg. (KKU); • 7 nymphs, **Khon Kaen Prov.**, Fisheries Pond KKU, 16°27'31.5"N, 102°48'38.3"E, 1.IV.2024, N. Tungpairojwong leg. (KKU); • 1 female imago, **Chiang Mai Prov.**, Mae Jam, 18°29'47.8"N, 98°21'38.3"E, 15.XII.2024, B. Boonsoong leg. (ZMKU); • 1 nymph, 1 male imago, **Phetchaburi Prov.**, Centara Life Cha-Am Beach Resort, 12°44'03.3"N, 99°57'52.3"E, 3.VII.2025, B. Boonsoong leg. (ZMKU); • 4 nymphs, **Surat Thani Prov.**, Makham Tia Subdistrict, 9°06'32.9"N, 99°22'35.0"E, 1.X.2025, S. Kwanboon leg. (ZMKU).

#### Description

**(in alcohol). Male imago** (Fig. 7B). See Navás (1923) (original description).

The male imagos of this species can be identified by their transparent wings (Fig. 11D). The abdominal terga are translucent white, with the posterior margins of segments I–VII marked with brown pigmentation. Segments II and V bear central reddish-brown to purple spots, while segments III and VI possess prominent reddish-brown to purple stripes along the lateral margins. Segments VIII–X are brown to rust-coloured (Fig. 14D). The abdominal sterna of segments VIII–IX and the posterior part of segment VII are white and lack distinct pigmentation (Figs 16D, 23B). The compound eyes are brown to rust-coloured, with olive-green lower portions (Fig. 9D).

**Female imago** (Fig. 7A): Body length 4.68–4.73 mm.

***Head***: Cream-white, with white to pale yellow midline flanked by two long orange to rust-coloured stripes covering the middle part of the head; upper margin white, with rust-coloured spots on the posterior part (Fig. 23A).

***Thorax***: Cream-white, with white to pale yellow midline flanked by two narrow, long, orange to rust-coloured stripes that extend from the head, bordered on outer that by narrow white lines. Lateral margins of anterior thorax feature rust-coloured stripes. Posterior part has darker brown stripes, connecting with the patterns of the abdominal segments (Fig. 23A).

***Legs***: Forelegs with femora that are predominantly rust-coloured (Fig. 9C).

***Abdominal terga***: Cream-white, with reddish-brown to red patterns. Segments II to IX, anterior part with a pair of pale spots or drop-shaped stripes, without a pale midline. The lateral margins of each anterior segment with reddish-brown to purple spots or streaks, which are larger and more distinct on segments III and VI. Segments VII to IX have dark midline, while segment X is rust-coloured (Fig. 14C).

***Abdominal sterna***: Cream-white with brittle white markings. Lateral margins at middle of sterna on segments II to VII with broad reddish-brown to red stripes along their entire length. Segments VI to IX with dark spots in the posterior part; posterior part of segment X is dark-coloured (Fig. 16C).

***Caudal filaments***: Two caudal filaments transparent white with dark purple joints (Fig. 9C).

***Wings***: Hyaline, with yellowish-golden veins and no dark bands. Pterostigmata area contains two straight veins (Fig. 11C).

**Mature nymphs (in alcohol)**: Body length 3.92–3.94 mm, terminal filaments 2.91–2.93 mm.

**Figure 4.**
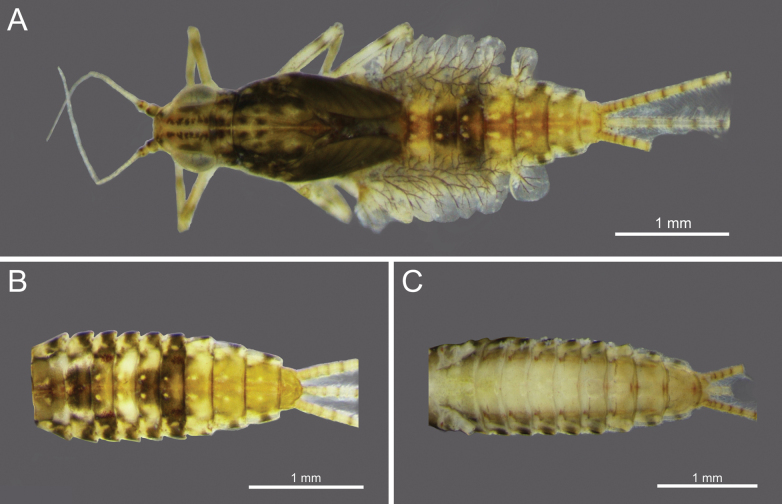
*Cloeon rubellum*, mature nymph morphology: **A.** Female mature nymph (dorsal view); **B.** Terga I–X; **C.** Sterna I–X.

***Head***: Two rows of irregular brown spots (Fig. 4A). Antennae mostly brown, with darker colouration at the base (Fig. 24A).

***Mouthparts***: i). Mandibles: Molar area contains densely distributed molar teeth. Lateral margins with sparsely distributed hair-like setae. Large prostheca, inner incisors, and outer incisors (Fig. 24B, C). ii). Labrum: Dorsal surface and half margins covered with hair-like setae. More than half of the surface contains sparsely distributed hair-like setae, with tufts of setae on either side of anterior notch (Fig. 24D). iii). Maxillary Palp: Segments I and II with sparsely distributed hair-like setae on the surface, while segment III has more widely distributed hair-like setae (Fig. 24E). iv). Labial Palpi: Segment I longer than segments II and III. Segments I and II with sparsely distributed hair-like setae, while segment III covered with hair-like setae that are longer and denser than those on other segments. Glossae with short setae along the margins, while paraglossae have longer setae on the margins (Fig. 24F).

***Thorax***: irregular brown markings (Fig. 4A).

***Legs***: Femora of all legs pale but with brown stripes near apex and base; short setae on surface and both margins. Tibiae and tarsi with brown bands near their bases, with short, sparsely distributed setae on the surface and dense, long setae along inner margins (Fig. 25A–C). Base of claws (foreleg) expanded, with basal half containing two rows of spines with gradation size (Fig. 25D).

***Abdominal terga***: Cream-white to pale brown. Segments III–IX, anterior part with very pale midline flanked by pair of pale spots or drop-shaped stripes. Segments II and III and V and VI may appear darker. Segments II and V (and occasionally III and VI) lack a pale midline, while segments IV and VII appear paler than other segments. Lateral margins of segments III and VI with large, prominent reddish-brown to purple stripes, similar to those of imago. Posterior half of lateral margins of segment VIII has four spines, while more than half of the lateral margin of segment IX has 5–6 spines (Figs 4A, B, 25E).

***Abdominal sterna***: Cream-white to pale brown, segments VI–IX with red to pale red spots in the posterior part (Fig. 4C).

***Caudal filaments***: Each segment with a ring of spines at the joints. Cerci with long setae along inner margins and large, long spines on outer margins. Terminal filament shorter than the cerci and long setae along both margins (Figs 4A, 25F).

**Egg.** Width 64.15 μm; Height 94.66 μm; granules ranging from 0.068–0.107 μm. Oval-shaped; surface covered with granules of varying irregular sizes and unevenly distributed across the surface (Fig. 23C, D).

#### Distribution.

Northern Thailand (Chiang Mai province); northeastern Thailand (Buri Ram, Khon Kaen, and Nakhon Ratchasima provinces); central Thailand (Bangkok and Lop Buri provinces); eastern Thailand (Chachoengsao and Chanthaburi provinces); western Thailand (Phetchaburi province); southern Thailand (Surat Thani province).

#### Remarks.

The male imago of *Cloeon rubellum* was described by Navás (1923) based on materials from the Philippines. Ours is the first record of this species in Thailand. In this study, we provide the first description of the female imago, egg structure, and nymph of *C. rubellum*.

### ﻿ Cloeon viridulum

Taxon classificationAnimaliaEphemeropteraBaetidae

Navás, 1931

EF08435E-D5E0-5263-9B18-021773E0758D

Figs 5A–C, 7D, 12A–D, 17A–D, 26A–D, 27E, 28D, 29B

Cloeon viridulum Navás, 1931: 7, fig. 14 (original description, female subimago).
Cloeon viridulum : Navás 1933: 17. Wu 1935: 251. Ulmer 1936: 215. Gui 1985: 82. Alba-Tercedor and Peters 1985: 221. Sun et al. 2015: 58 (habitat). Si et al. 2017:1 (transcriptome). Ying et al. 2021: 12 (C. virens described by Ulmer (1925, 1936) was synonymised with C. viridulum).

#### Material examined.

• 2 female imagos, **Nakhon Ratchasima Prov.**, Motorway M6, 14°53'09.8"N, 101°37'17.5"E, 19.V.2025, B. Boonsoong leg. (ZMKU); • 1 nymph, 3 male imagos, 2 female imagos, **Kanchanaburi Prov.**, The River Kwai Bridge Resort, 14°02'26.7"N 99°30'26.6"E, 05.VII.2025, B. Boonsoong leg. (ZMKU).

#### Description

**(in alcohol). Female subimago.** See Navás (1931) (original description).

The female imagos are characterised by a green to yellowish-green head, with a pale-yellow midline flanked by two dark brown longitudinal stripes across the central region (Fig. 26A). The thorax is yellowish-green, with a yellowish-green midline bordered by two narrow, long, pale brown longitudinal stripes extending from the head (Fig. 26A). The abdominal terga are yellowish-green, with uniformly brown to dark reddish-brown pigmentation. Terga II–VIII have a pale midline flanked by a pair of pale spots or drop-shaped markings, while the lateral margins of each anterior segment bear dark reddish-brown spots or short stripes (Fig. 17A). The abdominal sterna exhibit dark reddish-brown spots along the lateral margins of segments II–VIII (Fig. 17C). The wings are hyaline, with yellowish-green to translucent yellow venation in the costal and subcostal areas (Fig. 12C).

**Figure 5.**
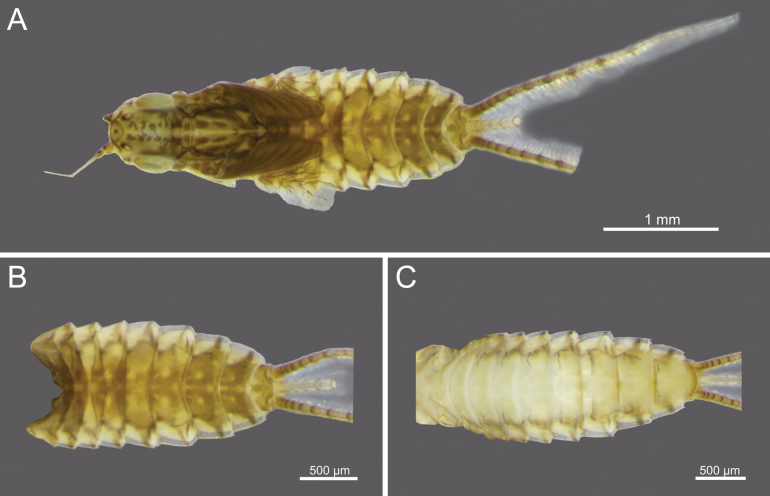
*Cloeon viridulum*, mature nymph morphology: **A.** Female mature nymph (dorsal view); **B.** Terga I–X; **C.** Sterna I–X.

**Figure 6.**
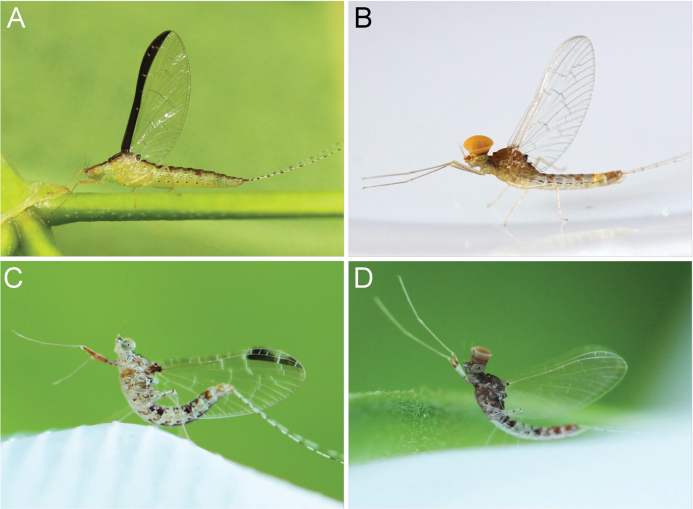
Live habitus of imago of *Cloeon* species in Thailand: **A.** Female of *Cloeon bengalense*; **B.** Male of *Cloeon bengalense*; **C.** Female of *Cloeon harveyi*; **D.** Male of *Cloeon harveyi*.

**Figure 7.**
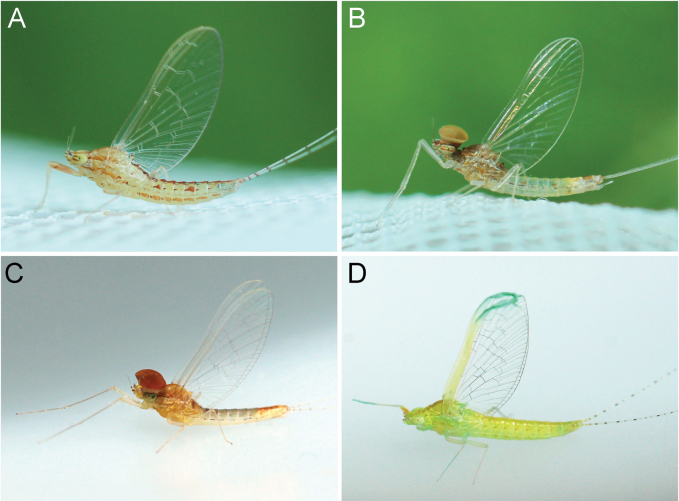
Live habitus of imago of *Cloeon* species in Thailand: **A.** Female of *Cloeon rubellum*; **B.** Male of *Cloeon rubellum*; **C.** Male of *Cloeon bicolor*; **D.** Female of *Cloeon viridulum*.

**Figure 8.**
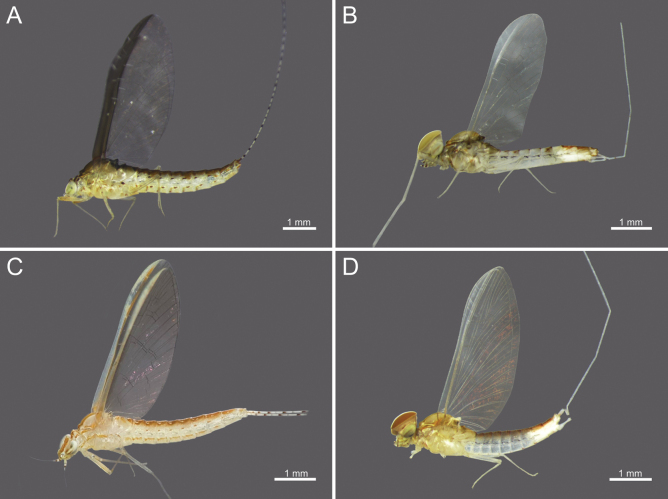
Adult habitus of *Cloeon* species in Thailand: **A.** Female of *Cloeon bengalense*; **B.** Male of *Cloeon bengalense*; **C.** Female of *Cloeon bicolor*; **D.** Male of *Cloeon bicolor*.

**Figure 9.**
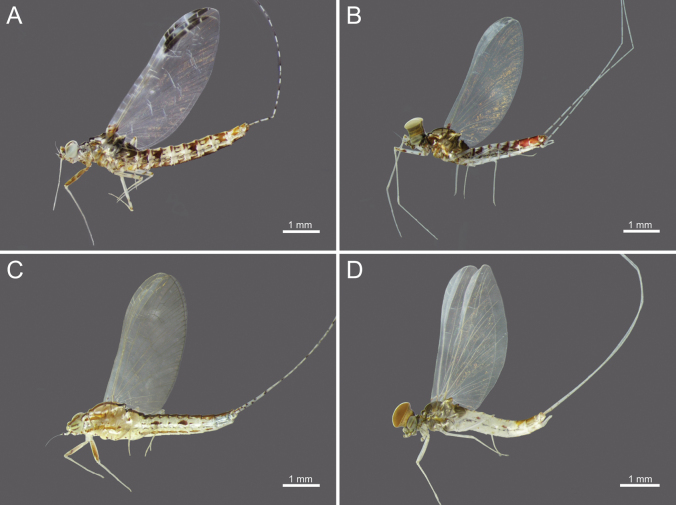
Adult habitus of *Cloeon* species in Thailand: **A.** Female of *Cloeon harveyi*; **B.** Male of *Cloeon harveyi*; **C.** Female of *Cloeon rubellum*; **D.** Male of *Cloeon rubellum*.

**Figure 10.**
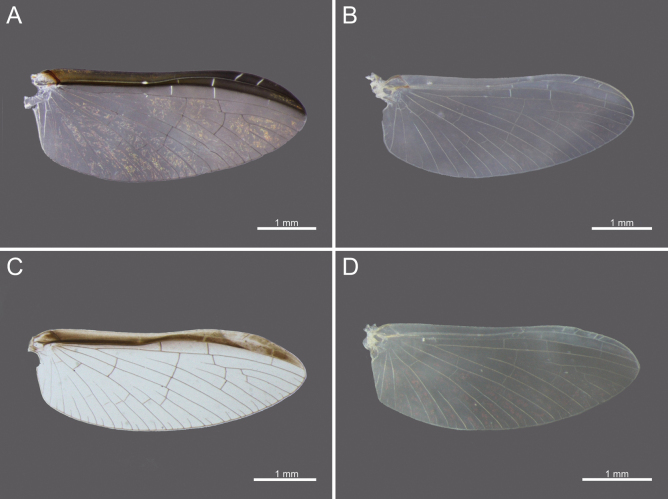
Wings of *Cloeon* species in Thailand: **A.** Female of *Cloeon bengalense*; **B.** Male of *Cloeon bengalense*; **C.** Female of *Cloeon bicolor*; **D.** Male of *Cloeon bicolor*.

**Figure 11.**
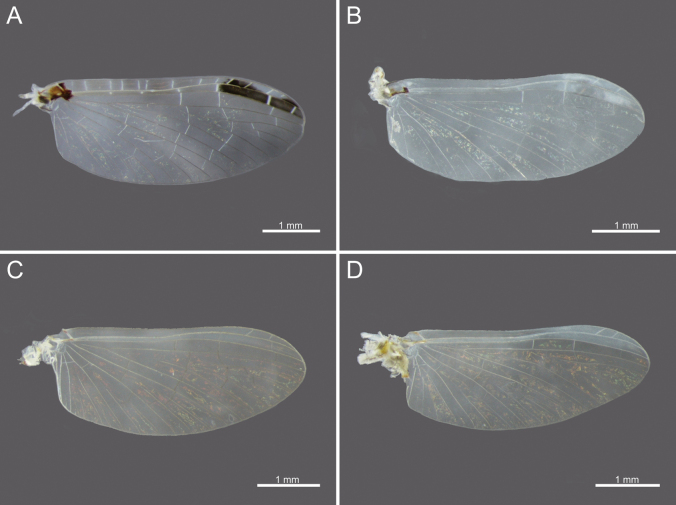
Wings of *Cloeon* species in Thailand: **A.** Female of *Cloeon harveyi*; **B.** Male of *Cloeon harveyi*; **C.** Female of *Cloeon rubellum*; **D.** Male of *Cloeon rubellum*.

**Figure 12.**
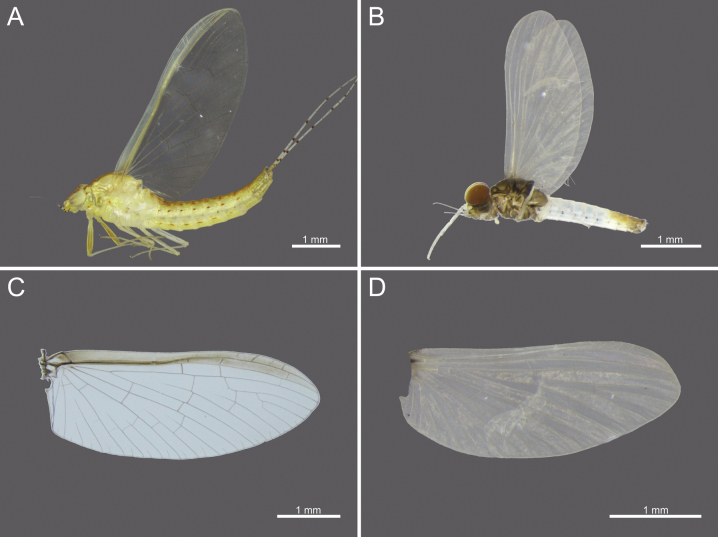
External morphology of *Cloeon viridulum* in Thailand: **A.** Female imago; **B.** Male subimago; **C.** Wing of female imago; **D.** Wing of male subimago.

**Figure 13.**
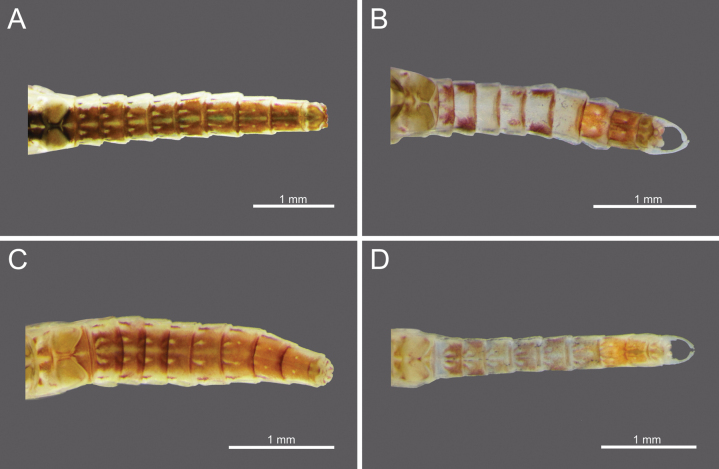
Adult abdominal terga of *Cloeon* species in Thailand: **A.** Female of *Cloeon bengalense*; **B.** Male of *Cloeon bengalense*; **C.** Female of *Cloeon bicolor*; **D.** Male of *Cloeon bicolor*.

**Figure 14.**
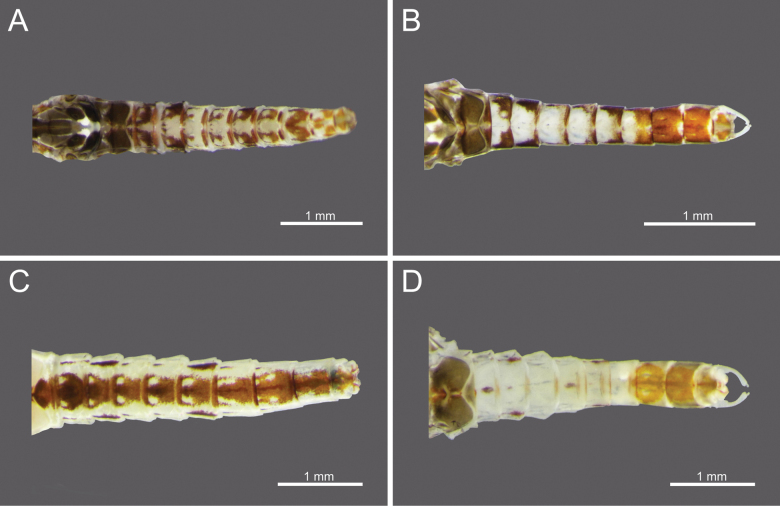
Adult abdominal terga of *Cloeon* species in Thailand: **A.** Female of *Cloeon harveyi*; **B.** Male of *Cloeon harveyi*; **C.** Female of *Cloeon rubellum*; **D.** Male of *Cloeon rubellum*.

**Figure 15.**
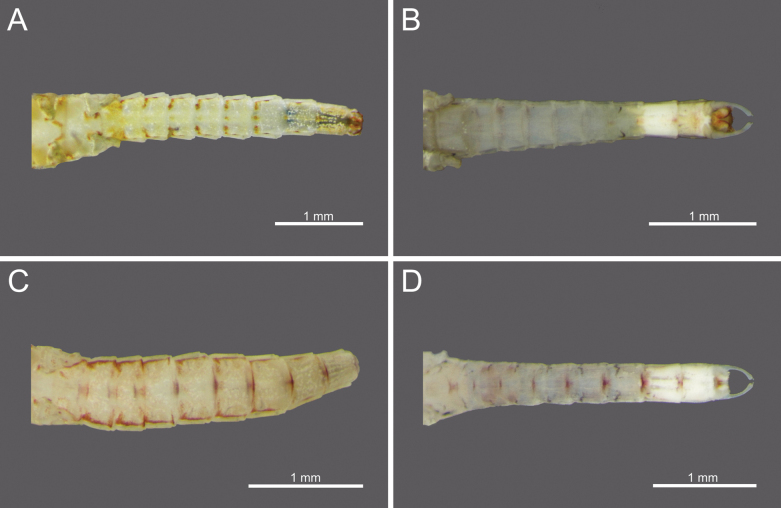
Adult abdominal sterna of *Cloeon* species in Thailand: **A.** Female of *Cloeon bengalense*; **B.** Male of *Cloeon bengalense*; **C.** Female of *Cloeon bicolor*; **D.** Male of *Cloeon bicolor*.

**Figure 16.**
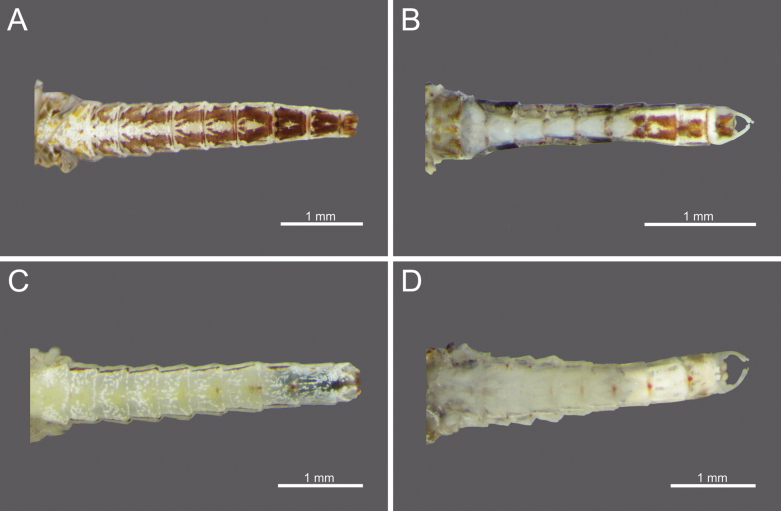
Adult abdominal sterna of *Cloeon* species in Thailand: **A.** Female of *Cloeon harveyi*; **B.** Male of *Cloeon harveyi*; **C.** Female of *Cloeon rubellum*; **D.** Male of *Cloeon rubellum*.

**Figure 17.**
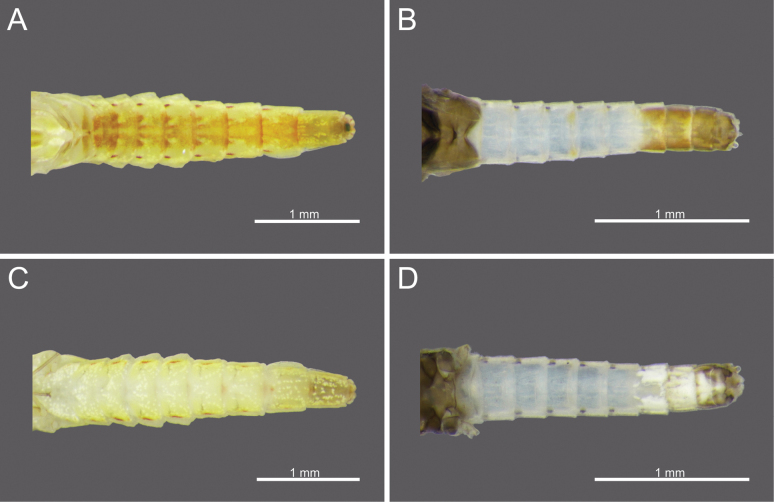
Adult abdominal morphology of *Cloeon viridulum* in Thailand: **A.** Abdominal terga of female imago; **B.** Abdominal terga of male subimago; **C.** Abdominal sterna of female imago; **D.** Abdominal sterna of male subimago.

**Figure 18.**
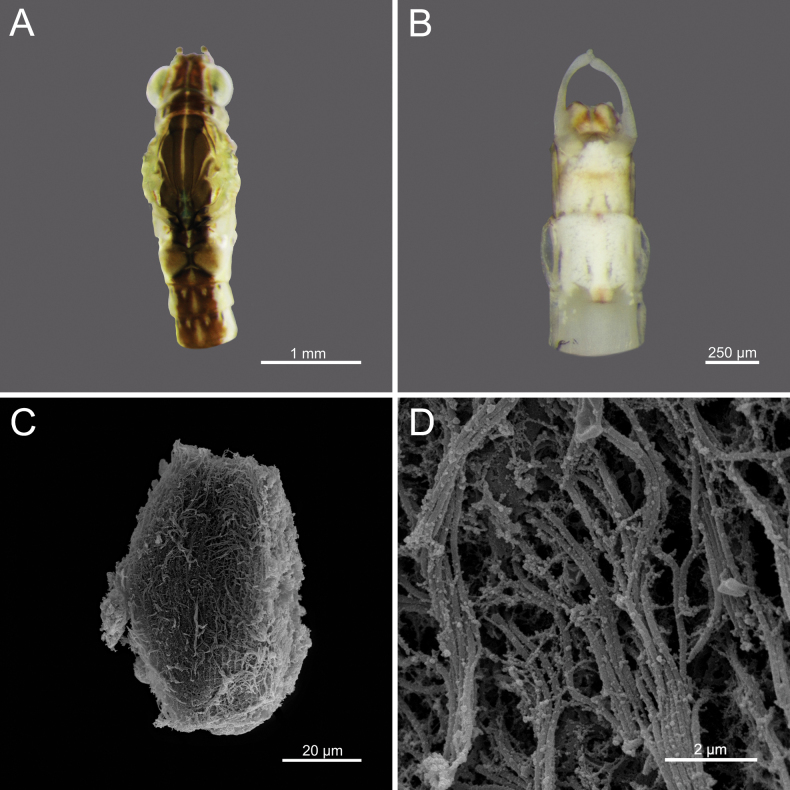
*Cloeon bengalense*, adult morphology: **A.** Head and thorax of female imago (dorsal view); **B.** Sterna VIII–X of male imago (ventral view), SEMs of egg structures: **C.** General outline of egg; **D.** Chorion surface.

The mature nymphs possess a head with two rows of irregular brown spots (Fig. 5A). The abdominal terga are dark green to brown, with uniformly dark brown to brown colouration. Terga II–IX bear a pale midline flanked by a pair of pale spots or drop-shaped marks. The abdominal sternum X lacks dark pigmentation (Fig. 5C).

**Male subimago (in alcohol).** Body length 3.05–3.11 mm.

***Head***: Dark brown. Eyes orange to rust-coloured with olive green lower eyes (Fig. 12B).

***Thorax***: Olive green to dark brown, with white to cream-white midline (Fig. 12B).

**Figure 19.**
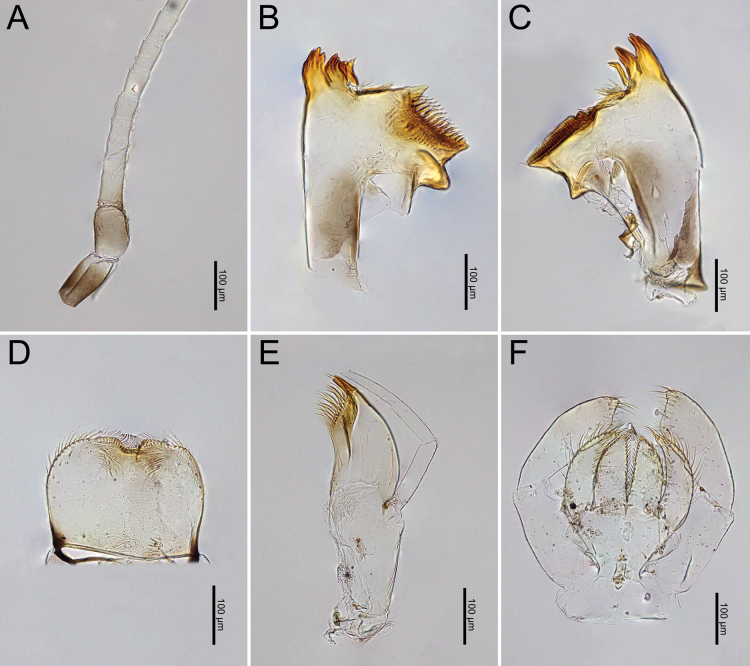
*Cloeon bengalense*, mature nymph morphology: **A.** Antenna; **B.** Left mandible; **C.** Right mandible; **D.** Labrum; **E.** Maxilla; **F.** Labium.

***Legs***: White colouration (Fig. 12B).

***Abdominal terga***: White; posterior parts of segments II–VI bear a pale pair of rust-coloured stripes. Lateral margins of segments II–VII anteriorly with dark reddish-purple spots or stripes. Segments VII–X rust-coloured (Fig. 17B).

***Abdominal sterna***: White; lateral margins of segments II–VII anteriorly have dark reddish-purple spots or stripes. Segments VII–X white (Fig. 17D).

***Wings***: Milky white, with cream-white veins and no dark bands. Posterior margin with sparse microtrichia (Fig. 12D).

**Egg.** Width 49.78 μm; height 84.99 μm. Oval-shaped; surface covered with pits, with irregularly distributed depressions of varying sizes and irregular dimensions and unevenly distributed across the surface (Fig. 26C, D).

#### Distribution.

Northeastern Thailand (Nakhon Ratchasima province); western Thailand (Kanchanaburi province).

#### Remarks.

The female subimago of *Cloeon viridulum* was described by Navás (1931) based on materials from China. Ours is the first record of this species in Thailand. In this study, we provide the first description of the male subimago and egg structure of *C. viridulum*.

**Figure 20.**
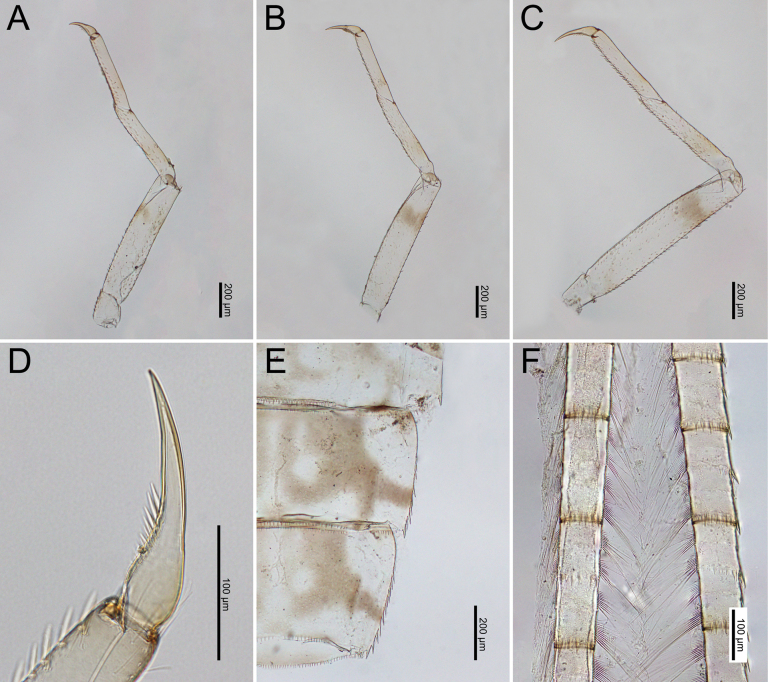
*Cloeon bengalense*, mature nymph morphology: **A.** Foreleg; **B.** Midleg; **C.** Hindleg; **D.** Claw of foreleg; **E.** Lateral margins of Terga VIII–IX; **F.** Partial of caudal filaments.

##### ﻿Molecular analysis

The partial sequence of the mitochondrial *COI* gene (580–660 bp) of Thai *Cloeon* species (Table 1) was analysed and compared with the sequences of *C. viridulum* (?), *C. dipterum*, *C. navasi*, and *Cloeon* sp. retrieved from GenBank. The phylogenetic tree was inferred using the Maximum Likelihood (ML) method. The 36 sequences separated clades as shown in Fig. 30. The first clade included three *C. viridulum* ? (China) and the second, one *C. viridulum* (Thailand); the third, *C. bicolor* (Thailand); the fourth, *C. bengalense* (Thailand); the fifth, *C. rubellum* (Thailand) and an unknown *Cloeon* sp. from Thailand; the sixth, *C. harveyi* (Thailand) and *C. navasi* (China); and the seventh, *C. dipterum* (USA, Japan, and Germany).

Similarly, species delimitation analysis using the ASAP method supported the separation of clades 1–6. The ASAP analysis grouped *C. rubellum* and *Cloeon* sp., as well as *C. harveyi* (Thailand) and *C. navasi* (China), as a single species. Interestingly, in clade 7, the ASAP analysis separated *C. dipterum* into three distinct groups: *C. dipterum* (USA), *C. dipterum* (Japan), and *C. dipterum* (Germany) (Fig. 30).

The genetic distance analysis revealed that the range of intraspecific genetic distances of the genus *Cloeon* from Thailand was 0–2.21%, while the range of interspecific genetic distances was 8.51–14.80% (Table 2). The genetic distances between *Cloeon viridulum* ? (China) and *C. viridulum* (Thailand) were higher (4.63–4.94%) than the maximum intraspecific distance. These results suggest that the two clades may represent distinct species. The interspecific distances between *C. viridulum* ? (China) and *C. bicolor* (Thailand) ranged 6.48–7.20%. In contrast, *C. viridulum* (Thailand) exhibited greater genetic distances from *C. bicolor* (Thailand) (8.51–8.82%).

**Table 2.** Genetic distance (*COI*) between sequenced specimens using the p-distance.

	Species	1	2	3	4	5	6	7	8	9	10	11
1	*Securiops primasia*	-										
2	*C. viridulum* ? (China)	14.81–14.97%	0.15–0.46%									
3	*C. dipterum* (USA)	13.53%	15.12–15.28%									
4	*C. dipterum* (Germany)	14.13%	14.81–14.97%	7.14%								
5	*C. dipterum* (Japan)	14.44%	14.66–14.81%	6.53%	8.05%							
6	*C. navasi* (China)	12.50%	12.46–12.77%	13.73–13.89%	13.73–13.89%	13.73–13.89%	0.91%					
7	*Cloeon* sp. (Thailand)	16.74%	12.60–12.76%	16.28%	16.74%	15.50%	11.81–11.97%					
8	*C. bengalense* (Thailand)	14.43–15.35%	11.88–13.18%	15.50–16.65%	15.50–16.56%	15.05–15.82%	12.33–13.50%	11.97–12.87%	0.32–1.97%			
9	*C. bicolor* (Thailand)	16.87–17.32%	6.48–7.20%	16.11–16.69%	16.72–17.17%	15.81–16.22%	13.89–14.40%	13.95–14.80%	12.92–14.56%	0.31–1.37%		
10	*C. harveyi* (Thailand)	12.31–12.77%	12.65–13.27%	13.83–14.29%	13.07–14.13%	13.68–14.22%	0.46–1.08%	12.25–12.56%	11.99–13.77	13.55–14.49%	0.15–1.22%	
11	*C. rubellum* (Thailand)	15.65–16.56%	11.72–12.82%	15.35–15.93%	16.11–17.51%	14.44–15.34%	11.27–12.66%	0.47–2.21%	11.15–13.09%	13.62–14.65%	11.55–12.93%	0–1.82%
12	*C. viridulum* (Thailand)	15.35%	4.63–4.94%	17.93%	17.48%	16.57%	12.50–12.96%	14.26%	12.62–13.77%	8.51–8.82%	13.24–13.83%	12.59–14.29%

Both the *Cloeon bicolor* (Thailand) clade and the *C. bengalense* (Thailand) clade showed the expected intraspecific distance ranges of 0.31–1.37% and 0.32–1.97%, respectively. The unknown *Cloeon* sp. from Thailand exhibited genetic distances closest to the *C. rubellum* (Thailand) clade (0.47–2.21%). Similarly, *C. navasi* (China) showed genetic distances closest to the *C. harveyi* (Thailand) clade (0.46–1.08%), both of which fell within the range of the intraspecific genetic distances in Thailand (0–2.21%). Interestingly, the *C. dipterum* clade showed a relatively high range of intraspecific genetic distances (6.53–8.05%), which was higher than the intraspecific range found in Thailand but remained below the minimum interspecific distance (8.51%).

### ﻿Key to species of the genus *Cloeon* in Thailand

#### ﻿Eggs

**Table:** 

1	Egg surface overall covered with long filamentous structures	***C. bengalense* (Fig. 18C, D)**
–	Egg surface without long filamentous structures	**2**
2	Egg surface covered with depressions	***C. viridulum* (Fig. 26C, D)**
–	Egg surface covered without depressions	**3**
3	Egg surface covered with granules of consistent size and distribution, high density	***C. harveyi* (Fig. 22C, D)**
–	Egg surface covered with granules unevenly distributed	**4**
4	Egg surface covered with granules of similar size, with more widely spaced granules	***C. bicolor* (Fig. 21C, D)**
–	Egg surface covered with granules of varying sizes, randomly distributed	***C. rubellum* (Fig. 23C, D)**

#### ﻿Mature nymphs

**Table:** 

1	Sterna with colour patterns	***C. harveyi* (Fig. 27A)**
–	Sterna without colour patterns	**2**
2	Terga III and VI with distinct reddish to purple stripes on lateral margins	***C. rubellum* (Fig. 27B)**
–	Terga III and VI without distinct reddish to purple stripes on lateral margins	**3**
3	Terga with a pale midline flanked by pale spots on terga II–IX	***C. bengalense* (Fig. 27C)**
–	Terga with a pale midline flanked by pale spots on a few segments	**4**
4	Terga IV and VII are pale	***C. bicolor* (Fig. 27D)**
–	Terga IV and VII are not pale	***C. viridulum* (Fig. 27E)**

#### ﻿Adult females

**Table:** 

1	Wings without patterns or colouration along the margins	***C. rubellum* (Fig. 28A)**
–	Wings with patterns or colouration along the margins	2
2	Wings with dark brown to black markings at the pterostigma area	***C. harveyi* (Fig. 28B)**
–	Wings with brown to dark brown colouration along the entire margins	3
3	Margins of wings pale yellow at the costal area and dark brown at the subcostal area	***C. bicolor* (Fig. 28C)**
–	Margins of wings are same colour at the costal and subcostal areas	**4**
4	Margins of wings are yellowish-green at both the costal and subcostal areas, thorax with brown patterns and white-bordered stripes	***C. viridulum* (Fig. 28D)**
–	Margins of wings are dark brown at both the costal and subcostal areas; thorax uniformly dark brown	***C. bengalense* (Fig. 28E)**

#### ﻿Adult males

**Table:** 

1	Sterna VII–IX with colour patterns	***C. harveyi* (Fig. 29A)**
–	Sterna VII–IX without colour patterns	**2**
2	Terga II–VI without colour patterns	***C. viridulum* (Fig. 29B)**
–	Terga II–VII with colour patterns	**3**
3	Terga II–VII with consistent reddish-brown stripes	***C. bicolor* (Fig. 29C)**
–	Terga II–VII with stripes only on some segments	**4**
4	Terga III and VI with dark reddish-purple stripes that extend nearly to the lateral margins, with smaller streaks on terga II and V	***C. bengalense* (Fig. 29D)**
–	Terga III and VI with prominent and large reddish-brown to purple stripes on the lateral margins	***C. rubellum* (Fig. 29E)**

## ﻿Discussion

This study demonstrated that the genus *Cloeon* is commonly found in lentic habitats in Thailand. Five species were identified—*C. bengalense*, *C. bicolor*, *C. harveyi*, *C. rubellum* and *C. viridulum*—based on morphological, molecular, and taxonomic analyses. In Thailand, the five species coexist randomly in various lentic habitats, ranging from small temporary wetlands to large freshwater ponds. Our analysis indicates that *C. harveyi* is a common species, with the widest distribution in Thailand. Additionally, this study provides the first record of *C. bengalense*, *C. rubellum and C. viridulum* in the country.

**Figure 21.**
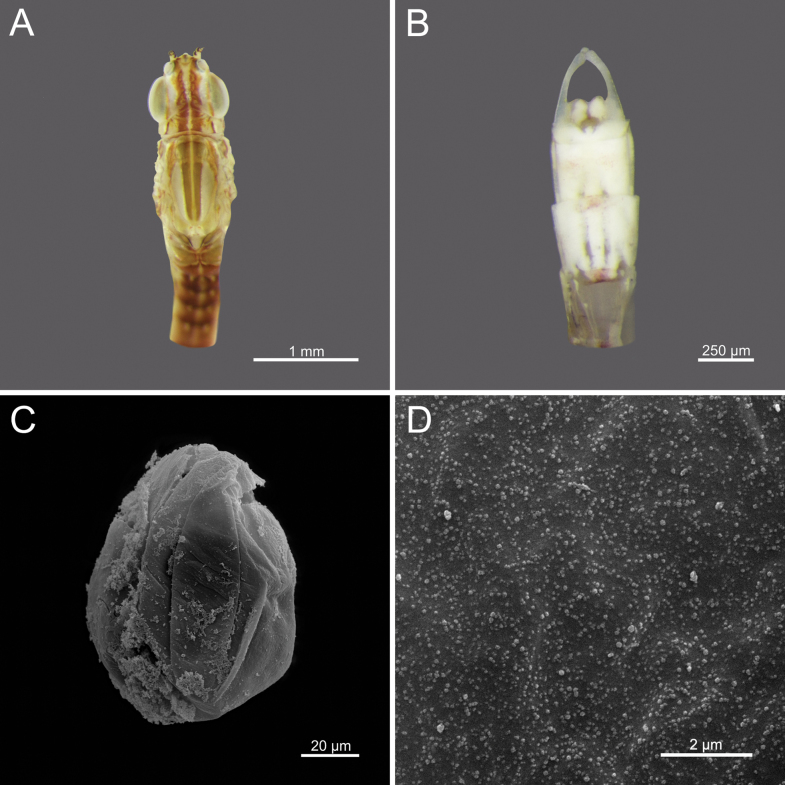
*Cloeon bicolor*, Adult morphology: **A.** Head and thorax of female imago (dorsal view); **B.** Sterna VIII–X of male imago (ventral view), SEMs of egg structures: **C.** General outline of egg; **D.** Chorion surface.

**Figure 22.**
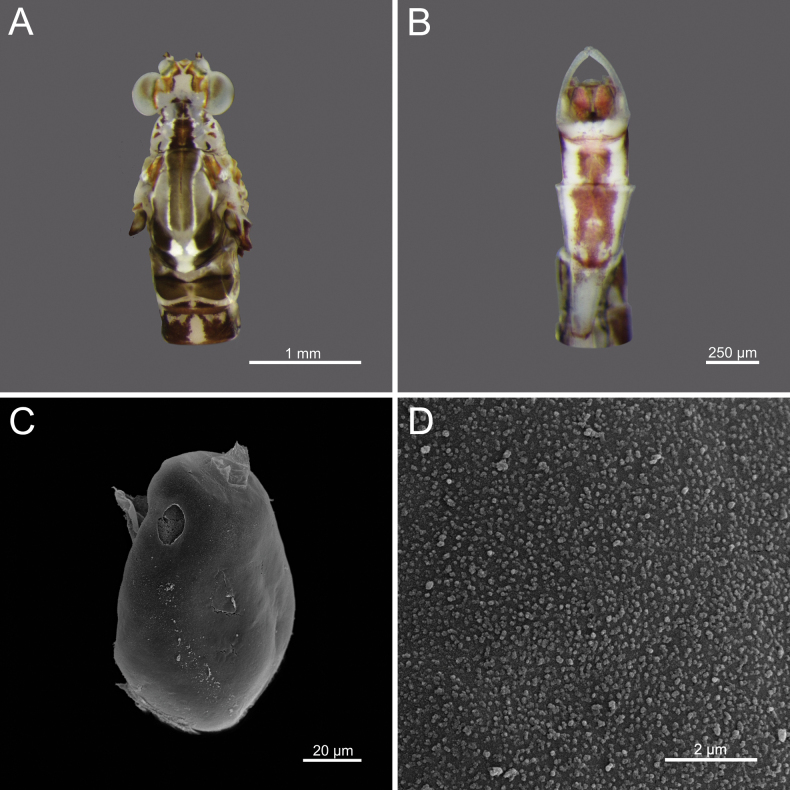
*Cloeon harveyi*, Adult morphology: **A.** Head and thorax of female imago (dorsal view); **B.** Sterna VIII–X of male imago (ventral view), SEMs of egg structures: **C.** General outline of egg; **D.** Chorion surface.

The mature nymph and adult stages of *Cloeon harveyi* can be readily identified by two reddish-brown stripes fused on the terminal sterna. In addition, the mature nymphs of *C. rubellum* are easily recognised by large distinct markings on terga III and VI while imagos of *C. rubellum* bear prominent reddish-brown stripes on the lateral margins of terga III and VI. In *Cloeon bengalense*, the lateral margins of terga III and VI exhibit dark reddish-brown spots or short stripes extending almost to the lateral margins, and the female imago has dark brown venation in the costal and subcostal areas of the wings. In *Cloeon bicolor*, the lateral markings on terga III and VI do not extend to the lateral margins, and the female imago has a costal area with a pale-yellow stripe and a subcostal area with a dark-brown stripe. The nymphs of *Cloeon viridulum* of Thai specimens differ from those of *Cloeon viridulum* (?) reported by Ying et al. (2021, fig. 1H), which nymphs have a distinctive dark spot on tergites II and V. This character is the most prominent feature of *C. micki*, whether its nymph, subimago or imago of both sexes (Tong and Dudgeon 2021), but *C. viridulum* lacks such characters as described. In addition, the male subimago of *C. viridulum* has translucent whitish terga. Interestingly, the female imago exhibits a colour pattern on the wings similar to that of *C. bengalense*, but its thorax and abdominal terga patterns resemble those of *C. bicolor*. The latter species can be identified by its yellowish-green venation in the costal and subcostal areas and a thorax with a yellowish-green midline bordered by two narrow, long, pale brown, longitudinal stripes extending from the head. (Tables 3–5).

**Figure 23.**
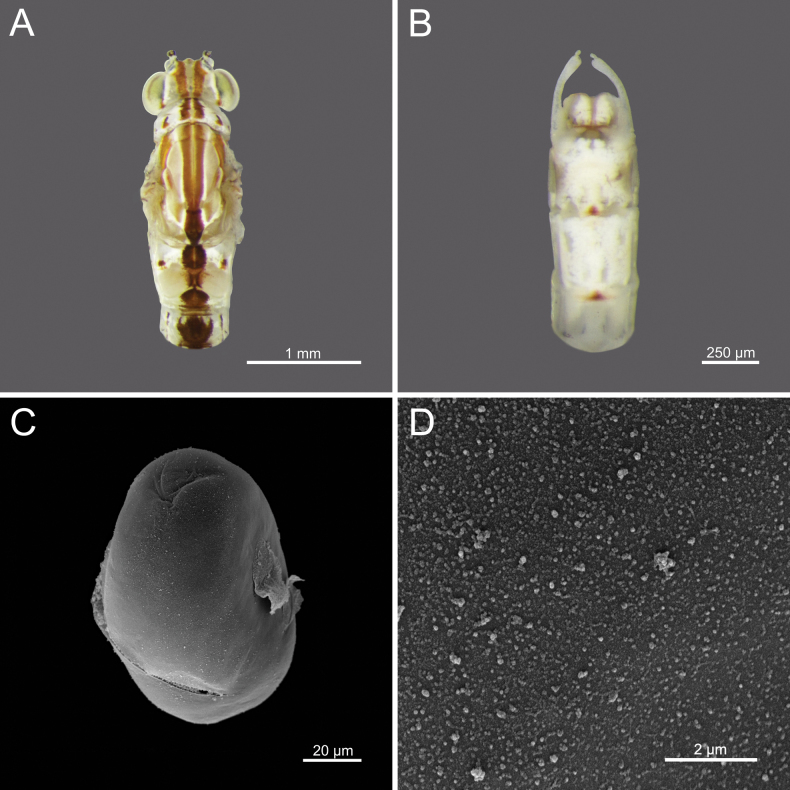
*Cloeon rubellum*, Adult morphology: **A.** Head and thorax of female imago (dorsal view); **B.** Sterna VIII–X of male imago (ventral view), SEMs of egg structures: **C.** General outline of egg; **D.** Chorion surface.

**Figure 24.**
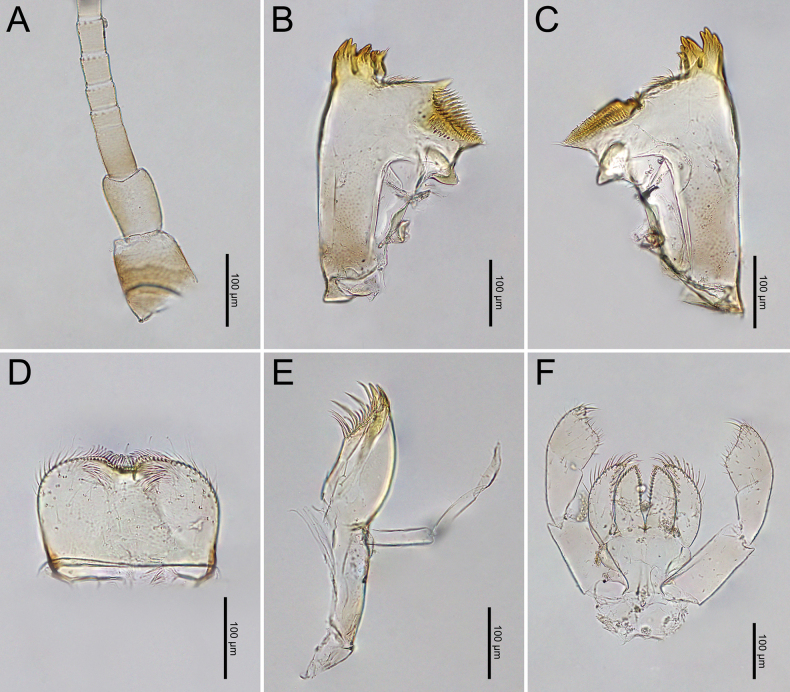
*Cloeon rubellum* mature nymph morphology: **A.** Antenna; **B.** Left mandible; **C.** Right mandible; **D.** Labrum; **E.** Maxilla; **F.** Labium.

**Table 3.** Comparison of mature nymph characteristics of the genus *Cloeon* in Thailand.

Characters	*C. bengalense*	*C. bicolor*	*C. harveyi*	*C. rubellum*	*C. viridulum*
Head pattern	Two rows of irregular brown spots.	Two rows of irregular brown spots.	Rust-coloured margin on the upper part.	Two rows of irregular brown spots.	Two rows of irregular brown spots.
Thorax pattern	irregular brown markings	irregular brown markings^b^	Amber brown^a^	Irregular brown markings	irregular brown markings
Abdominal terga pattern	Terga II–IX bear a pale midline flanked by a pair of pale spots or drop-shaped marks. Segments II and V may appear darker, while segments IV and VII may appear slightly paler but still retain the patterns.	Terga II–IX bear a pale midline flanked by a pair of pale spots or drop-shaped marks. Segments II and V may appear darker with interruptions in the midline.	Terga II, III, and VI with prominent reddish-brown patterns.	Terga III–IX, anterior part with a very pale midline flanked by a pair of pale spots or drop-shaped stripes. Segments II and V (and occasionally III and VI) lack a pale midline. The lateral margins of segments III and VI, large, prominent reddish-brown to purple stripes.	Terga II–IX bear a pale midline flanked by a pair of pale spots or drop-shaped marks.
Abdominal sterna pattern	Sterna X appears darker.	Sterna X lacks dark pigmentation.	Two reddish-brown stripes with a fuse on segments VIII–IX.	Sterna VI–IX with red to pale red spots in the posterior part.	Sterna X lacks dark pigmentation.

^a^Mukherjee et al. (2012); ^b^Ying et al. (2021).

**Table 4.** Comparison of female imago and egg characteristics of the genus *Cloeon* in Thailand.

Characters	*C. bengalense*	*C. bicolor*	*C. harveyi*	*C. rubellum*	*C. viridulum*
Head pattern	Pale green midline flanked by two dark brown longitudinal stripes covering most of the dorsal surface.	Pale-yellow midline flanked by two dark brown longitudinal stripes across the central region.	Rust-coloured margin on the upper part.	Pale yellow midline flanked by two long rust-coloured stripes covering the middle part of the head, the upper margin is white.	Yellowish-green midline flanked by two dark brown longitudinal stripes across the central region.
Thorax pattern	Yellowish-green with a pale midline flanked by two continuous dark brown longitudinal stripes extending from the head to the abdomen.	Yellow midline bordered by two narrow, long, pale brown longitudinal stripes extending from the head.	Pair of midline dark brown stripe that narrows toward the anterior, flanked by white lines.	Pale yellow midline flanked by two narrow, long, rust-coloured stripes that extend from the head, and posterior part has darker brown stripes that continue from the head to the abdomen	Yellowish-green midline bordered by two narrow, long, pale brown longitudinal stripes extending from the head.
Abdominal terga pattern	Terga II–IX have a pale midline flanked by a pair of pale spots or drop-shaped stripes; the lateral margins of anterior terga exhibit dark reddish-brown spots or short stripes, with those on segments III and VI extending almost to the lateral margins.	Terga II–IX have a pale midline flanked by a pair of pale spots or drop-shaped markings, while the lateral margins of each anterior segment bear dark reddish-brown spots or stripes.	Terga II, III, and VI have prominent red-brown markings and posterior parts of terga I–IX bearing a pair of reddish-brown stripes.	Terga II–IX, anterior part with a pair of pale spots or drop-shaped stripes. The lateral margins of each anterior segment with reddish-brown to purple spots or streaks, which are larger and more distinct on segments III and VI.	Terga II–VIII have a pale midline flanked by a pair of pale spots or drop-shaped markings, while the lateral margins of each anterior segment bear dark reddish-brown spots or stripes.
Abdominal sterna pattern	Sterna exhibit dark reddish-brown spots along the lateral margins of segments II–VIII.	Sterna display dark reddish-brown stripes along the lateral margins of segments II–VIII.	Two reddish-brown stripes along the abdomen, fuse on the apical segment.	Lateral margins of sterna on segments II to VII with broad reddish-brown to red stripes along their entire length.	Sterna exhibit dark reddish-brown spots along the lateral margins of segments II–VIII.
Wings	Dark brown venation in the costal and subcostal areas.	Costal area with a pale-yellow stripe, and the subcostal area with a dark brown stripe.	Distinct dark markings at the pterostigma area, the costal and subcostal areas have pale brown markings.	Costal and subcostal with clean.	Yellowish-green venation in the costal and subcostal areas.
Chorionic surface	Long filamentous structures.	Granules of similar size, distributed unevenly.	Granules of consistent size and distribution, high density.	Granules of varying sizes and unevenly distributed.	Varied-sized pits and unevenly distributed.

**Table 5.** Comparison of adult male characteristics of the genus *Cloeon* in Thailand.

Characters	*C. bengalense*	*C. bicolor*	*C. harveyi*	*C. rubellum*	C. viridulum*
Eyes colour	Orange-yellow with olive green lower eyes	Orange to orange-yellow with olive green lower eyes	Yellowish-brown with olive green lower eyes	Brown to rust-coloured, with olive green lower eyes.	Orange to rust-coloured with olive green lower eyes
Abdominal terga pattern	Terga III and VI bearing dark reddish-purple stripes extending nearly to the lateral margins; smaller paired stripes occur on segments II and V.	Terga II–VII, anterior part with a pale midline flanked by a pair of pale spots, narrow, and faint reddish-brown spots on the lateral margins.	Terga II, III, and VI; segment VII has paler patterns, and segments VIII–IX are reddish brown.	Terga II and V bear central reddish-brown to purple spots, while segments III and VI possess prominent reddish-brown to purple stripes along the lateral margins.	Posterior parts of segments II–XI bearing a pale pair of rust-coloured stripes and the lateral margins of segments II–XII anteriorly have dark reddish-purple spots or stripes.
Abdominal sterna pattern	Sterna VIII and IX are white, while sternum X ranges from orange to dark brown.	Sterna VIII–X are white and unpigmented.	Sterna VIII–IX and the posterior portion of segment VII are white, with reddish-brown striped patterns.	Sterna VIII–IX and the posterior part of segment VII are white.	Sterna II–XII anteriorly have dark reddish-purple spots or stripes.

* The specimens of males *C. viridulum* are in the subimago stage.

Previously, four species of *Cloeon* had been reported in Thailand: *C. bicolor* Kimmins, 1947, *C. bimaculatum* Eaton, 1885, *C. harveyi* (Kimmins, 1947), and *C. marginale* Hagen, 1858 (Gillies 1949; Uéno 1961). However, we did not find *C. bimaculatum* or *C. marginale*. According to Uéno (1961), *Cloeon bimaculatum* was recognised in the country from an adult male in Chiang Mai, and *C. marginale* was recognised from adults collected in Chiang Mai and Bangkok. Comparing the morphological characteristics of *Cloeon bimaculatum* described by Eaton (1885) and additional morphological characters by Kimmins (1947), the adult exhibits abdominal terga patterns very similar to those of *C. harveyi*, but with paler markings. In addition, the adult female’s wing venation differs in the inclination and intensity of the pattern in the pterostigmatic area. These variations could reflect intraspecific morphological differences and will require further molecular analysis for confirmation. Similarly, *Cloeon marginale* was originally described based on the subimago stage by Hagen (1858), with additional details on the adult stage provided by Eaton (1885). Notably, the characteristics of adult abdominal terga patterns and colouration on the female’s wing are similar to those of *C. viridulum* from this study. This raises the possibility that *Cloeon bimaculatum* and *C. marginale*, which have been recorded from Thailand (Uéno, 1961) may be misidentifications of *C. harveyi* and *C. viridulum*, respectively.

**Figure 25.**
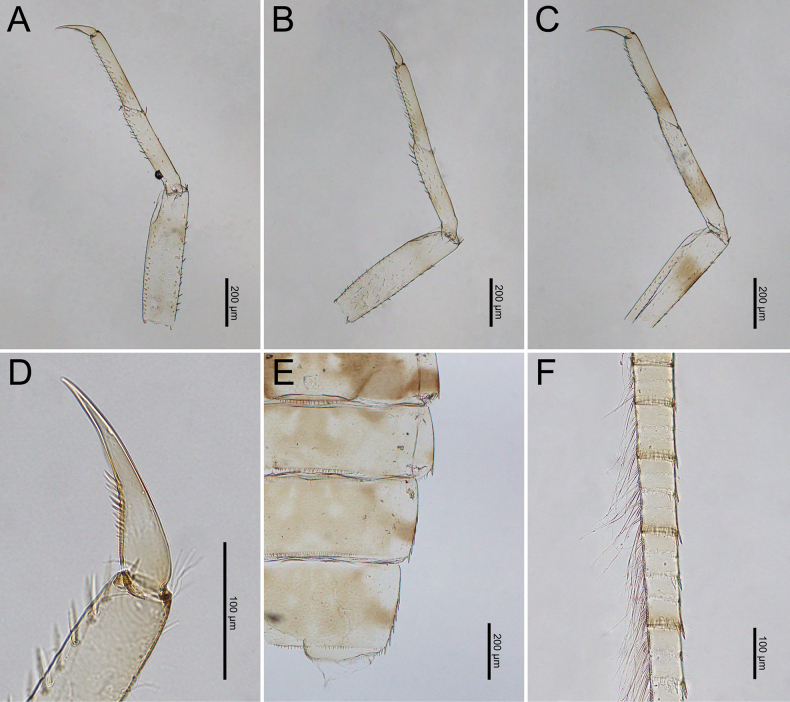
*Cloeon rubellum*, mature nymph morphology: **A.** Foreleg; **B.** Midleg; **C.** Hindleg; **D.** Claw of foreleg; **E.** Lateral margins of Terga VIII–IX; **F.** Partial of cerci.

**Figure 26.**
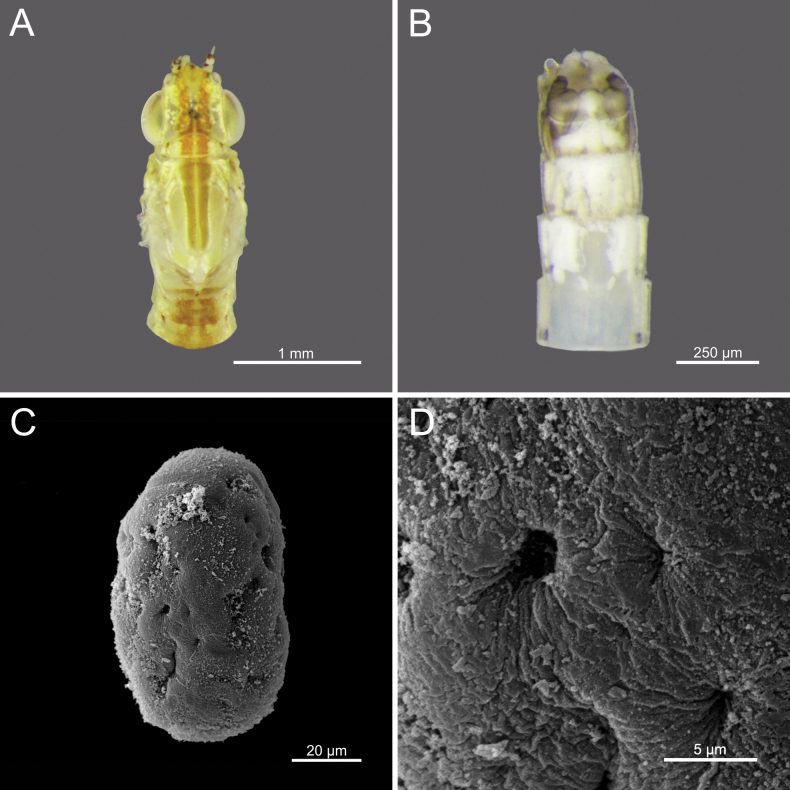
*Cloeon viridulum*, Adult morphology: **A.** Head and thorax of female imago (dorsal view); **B.** Sterna VIII–X of male subimago (ventral view), SEMs of egg structures: **C.** General outline of egg; **D.** Chorion surface.

In Southeast Asia and adjacent regions, *Cloeon bengalense* is reported from India (Kimmins 1947) and Singapore (Ng 2019), *C. rubellum* from the Philippines (Navás 1923), and *C. viridulum* from China (Navás 1931) and India (Ying et al. 2021). The external morphological characteristics observed in the present study (e.g., body colouration, abdominal terga patterns, and wing colouration or markings) are consistent with the descriptions in these previous reports, and the distribution data from the surrounding countries further confirm that the occurrences reported here are the first recorded reports of these three species in Thailand. Gillies (1949) exclusively reported *C. bicolor* and *C. harveyi* from Bangkok in the past, so the present study expands the known distribution of *C. bicolor* to the eastern and northeastern regions and *C. harveyi* to the eastern, northeastern, northern, western, and southern regions of Thailand.

**Figure 27.**
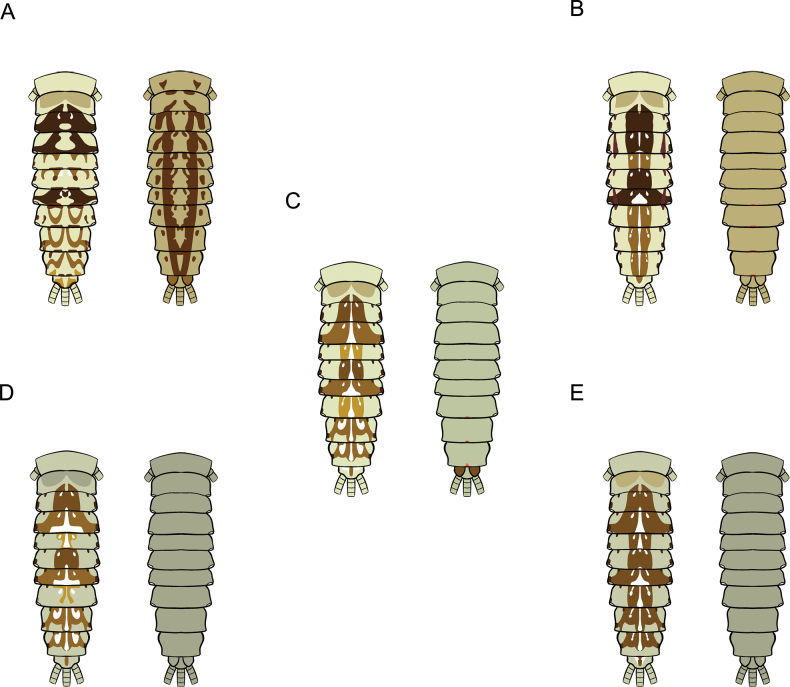
Drawing showing the dorsal (left) and ventral (right) views of a mature nymph *Cloeon* species in Thailand: **A.***Cloeon harveyi*; **B.***Cloeon rubellum*; **C.***Cloeon bengalense*; **D.***Cloeon bicolor*; **E.***Cloeon viridulum*.

The intraspecific distances of the genus *Cloeon* from Thailand (0–2.21%) are higher than the intra-group distances of *C. perkinsi* reported from Saudi Arabia, Israel, and Ethiopia (0.1–0.7%) (Yanai et al. 2020), but lower than the inter-group distances among populations from different countries (0.5–4.8%). These values are also comparable to the intraspecific distance of *C. peregrinator* (0.2%) (Benhadji et al. 2020). The interspecific distances (8.51–14.80%) obtained in this study are comparable to those previously reported (8.9–14%) (Benhadji et al. 2020; Yanai et al. 2020), and they are within the general range of interspecific variation, supporting the clear species separation of *Cloeon* in Thailand (Table 2).

**Figure 28.**
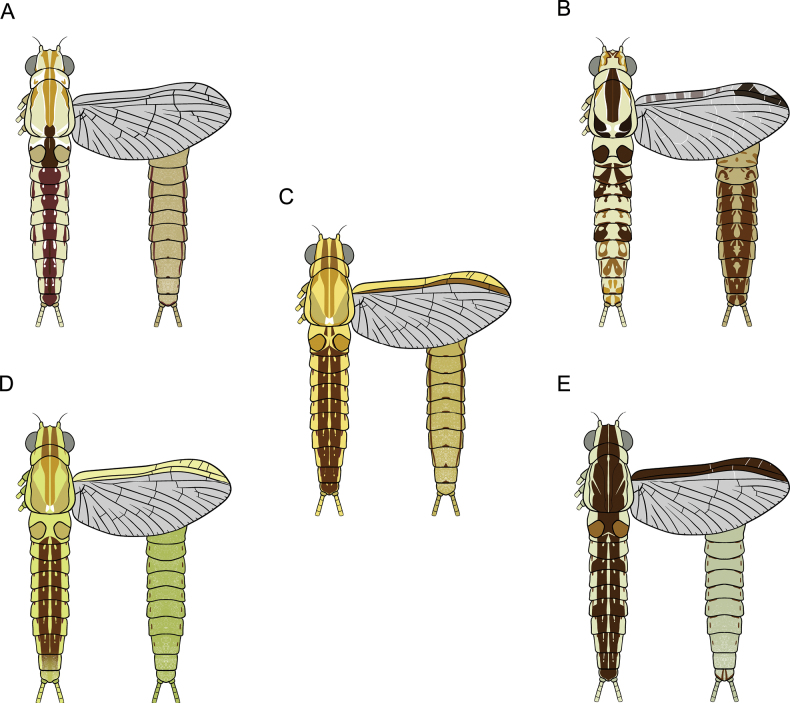
Drawing showing the dorsal (left) and ventral (right) views of a female imago of *Cloeon* species in Thailand: **A.***Cloeon rubellum*; **B.***Cloeon harveyi*; **C.***Cloeon bicolor*; **D.***Cloeon viridulum*; **E.***Cloeon bengalense*.

The sequencing data show that the interspecific distance between *Cloeon viridulum* ? (China) and *C. bicolor* (Thailand) (6.48–7.20%) is lower than the intraspecific distances of *C. dipterum* (6.53–8.05%). *Cloeon dipterum* has a wide range, and the sequences used in this study come from three different countries (the USA, Japan, and Germany) on three different continents. This large geographic distance could explain the relatively high intraspecific distances of *C. dipterum*. Previous studies have also noted taxonomic ambiguity between *Cloeon dipterum* and *C. peregrinator*, which are morphologically similar and remain difficult to identify (Benhadji et al. 2020). Moreover, morphological differences and colour patterns observed in mature nymphs, females, and males, together with molecular data, support the distinct separation between *C. viridulum* and *C. bicolor*, suggesting a possible recent divergence.

Both the ASAP method and genetic distance analysis confirm that *Cloeon viridulum* (Thailand) and *C. viridulum* (?) (China) represent different species. The *Cloeon* sp. (HM417037.1) from Thailand, previously known only from a photograph of a female imago showing morphological features identical to *C. rubellum*, is also identified as *C. rubellum*.

**Figure 29.**
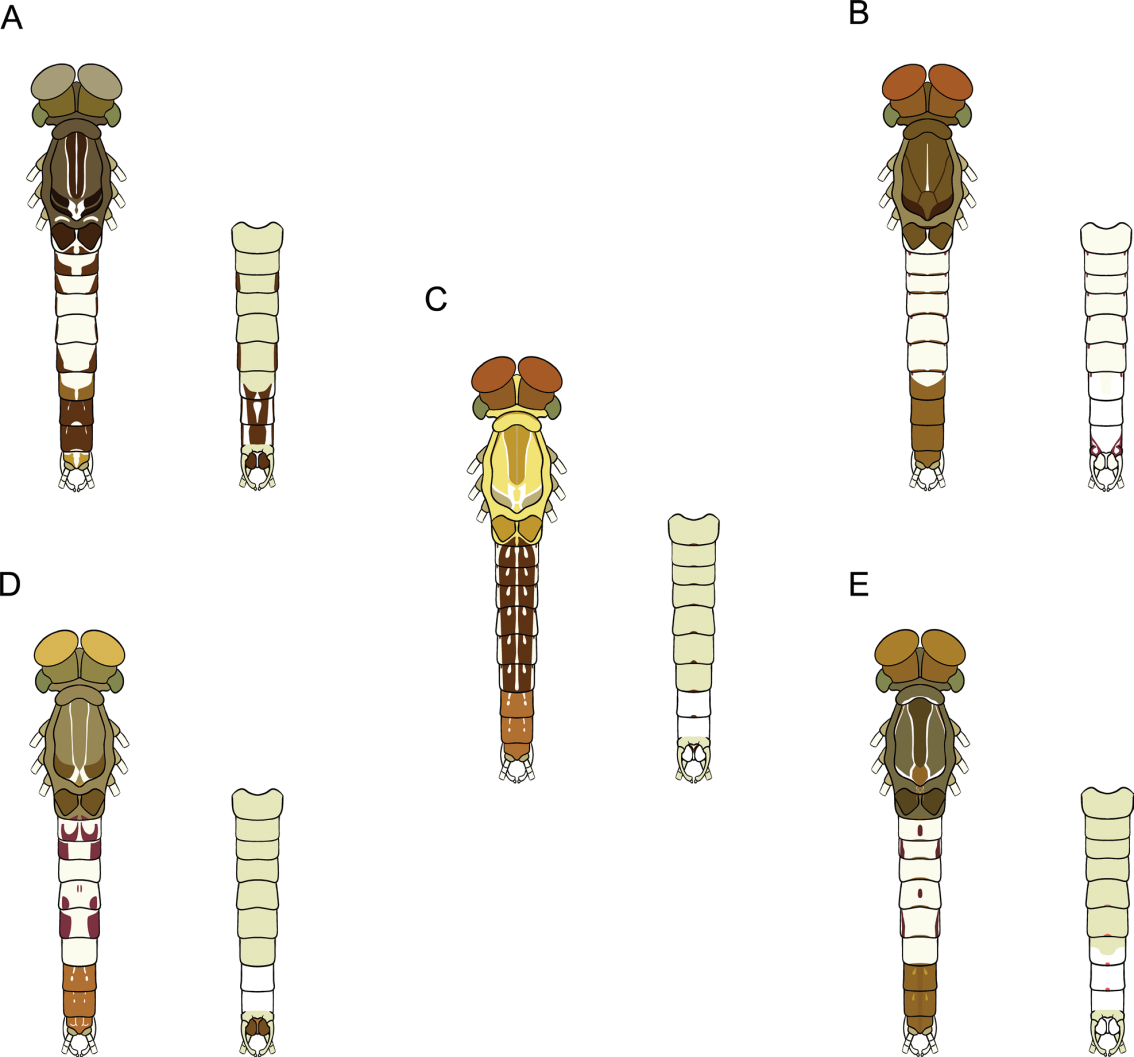
Drawing showing the dorsal (left) and ventral (right) views of a male adult of *Cloeon* species in Thailand: **A.***Cloeon harveyi* (imago); **B.***Cloeon viridulum* (subimago); **C.***Cloeon bicolor* (imago); **D.***Cloeon bengalense* (imago); **E.***Cloeon rubellum* (imago).

Similarly, *Cloeon navasi* (China) (KR612258.1, KR612259.1 from GenBank), which was originally described by Van Bruggen (1957) as possessing colourless wings, contrasts with *C. harveyi*, which is characterised by pigmented dark brown to black markings in the pterostigmatic area of the wing (Kimmins, 1947). According to Ying et al. (2021), *Cloeon navasi* is synonymised with *C. viridulum*. The present findings indicate that the two sequences of *Cloeon navasi* (China) represent a misidentification, and we identify them here as *C. harveyi*.

Our findings also indicate that the egg characteristics could be used for species identification of the genus *Cloeon* in Thailand. The chorionic surfaces of *Cloeon bicolor*, *C. harveyi* and *C. rubellum* are covered with granules, resembling those of *C. dipterum* (Ubero-Pascal et al. 2005). The egg surface of *Cloeon viridulum* is pitted, with irregularly distributed depressions, whereas the egg of *C. bengalense* has long filamentous structures, which may assist in adhesion (Table 4). Those structures may function like the knob-terminated coiled threads (KTCs) possessed by the genus *Afronurus*, which inhabits lotic waters and uses the KTCs to attach to substrates (Wongyam et al. 2023).

**Figure 30.**
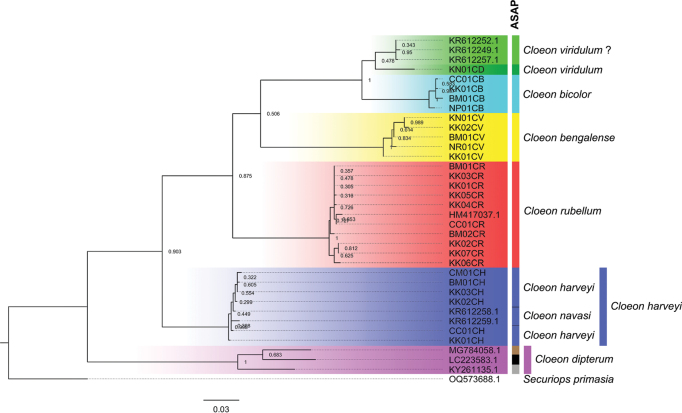
Phylogenetic tree of the genus *Cloeon* based on COI gene sequences inferred using the Maximum Likelihood (ML) method and performed using the distance-based Assemble Species by Automatic Partitioning (ASAP).

## Supplementary Material

XML Treatment for Cloeon bengalense

XML Treatment for Cloeon bicolor

XML Treatment for Cloeon harveyi

XML Treatment for Cloeon rubellum

XML Treatment for Cloeon viridulum

## ﻿Additional information

### Use of AI

No use of AI was reported.

### Author ORCIDs

Chayanon Noenrimnong https://orcid.org/0009-0005-9490-117X

Chanaporn Suttinun https://orcid.org/0000-0002-0380-1554

Nisarat Tungpairojwong https://orcid.org/0000-0001-8135-4246

Boonsatien Boonsoong https://orcid.org/0000-0002-8166-0021

### Data availability

All of the data that support the findings of this study are available in the main text.
